# Neoadjuvant chemoimmunotherapy for locally advanced esophageal squamous cell carcinoma: Data from literature review and a real‐world analysis

**DOI:** 10.1111/1759-7714.15291

**Published:** 2024-03-26

**Authors:** Yao Zhang, Huiting Li, Bo Yu, Si Sun, Zhihuang Hu, Xianghua Wu, Yang Zhang, Bin Li, Yawei Zhang, Jiaqing Xiang, Jialei Wang, Hui Yu

**Affiliations:** ^1^ Department of Medical Oncology Fudan University Shanghai Cancer Center Shanghai China; ^2^ Department of Oncology Shanghai Medical College, Fudan University Shanghai China; ^3^ Department of Radiation Oncology Fudan University Shanghai Cancer Center Shanghai China; ^4^ Department of Thoracic Surgery and State Key Laboratory of Genetic Engineering Fudan University Shanghai Cancer Center Shanghai China

**Keywords:** efficacy, locally advanced esophageal squamous cell carcinoma, neoadjuvant chemoimmunotherapy, safety, survival outcomes

## Abstract

**Background:**

Neoadjuvant chemoimmunotherapy (NCIT) for locally advanced esophageal squamous cell carcinoma (ESCC) is supported by increasing data, but the sample size is limited, and the findings are not completely consistent. We conducted a real‐world study and a meta‐analysis to evaluate the efficacy and safety of NCIT in locally advanced ESCC.

**Methods:**

We retrospectively assessed the outcomes of patients with locally advanced ESCC who completed NICT and subsequent esophagectomy at our hospital between January 2019 and December 2022, including pathological complete response (pCR) rate, major pathological response (MPR) rate, 1‐, 2‐, and 3‐year overall survival (OS) rates, disease control rate (DCR), objective response rate (ORR), 1‐year recurrence rate, R0 resection rate and adverse events. Moreover, a meta‐analysis of 27 published literatures was also conducted for comparison.

**Results:**

In the analysis, 128 patients were studied, with 25% achieving pCR, 46.1% MPR, and 99.2% R0 resection. The 1‐, 2‐, and 3‐year OS rates were 91.41% (95% CI: 85.15%–95.63%), 75.00% (95% CI: 66.58%–82.23%) and 64.84% (95% CI: 55.91%–73.07%).ORR and DCR were 31.2% (95% CI: 23.31–39.99) and 64.1% (95% CI: 55.15%–72.38%), and the 1‐year recurrence rate was 26.7% (95% CI: 22.5%–38.1%). Treatment‐related events occurred in 96.1% but were acceptable. In a meta‐analysis of 27 studies with 1734 patients, pooled rates for pCR, MPR, ORR, DCR, and R0 resection were 29%, 52%, 71%, 97%, and 98%, respectively, with a 1‐year recurrence rate of 12%.

**Conclusion:**

NCIT is safe and provides potential survival benefits for patients with locally advanced ESCC. However, randomized phase 3 trial data is still needed.

## Graphical abstract

The efficacy and safety of Neoadjuvant chemoimmunotherapy for locally advanced ESCC.

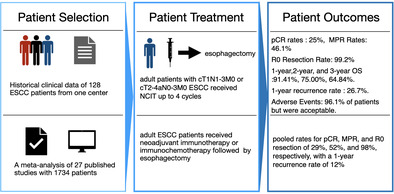

## INTRODUCTION

Esophageal cancer represents the sixth most common cancer worldwide, with an estimated 20 640 new cases and 16 510 deaths in 2022.^1^ The majority of this disease is histologically classified as squamous cell carcinoma and adenocarcinoma. Esophageal adenocarcinoma develops in a predominantly white, male population. In contrast, African Americans and Caucasian males have the most prevalence of esophageal squamous cell carcinoma (ESCC).^2^ About half of esophageal cancer patients have localized and regional disease, which includes spread to regional lymph nodes.

For patients with cT1N0M0 and cT2N0M0 lesions (low‐risk lesions), esophagectomy is the preferred treatment option. Patients with locally advanced thoracic esophageal or esophagogastric junction tumors who have not developed metastases to other organs opt for esophagectomy following neoadjuvant chemotherapy or chemoradiotherapy (NCT/NCRT).^3^ However, even with NCRT, the high incidence of postoperative recurrence and metastasis contributes to treatment failure.

With the application of antiprogrammed death‐(ligand) 1 (PD‐[L]1) antibodies in the treatment of ESCC shifting from the advanced to the perioperative setting, the clinical outcomes of patients have changed dramatically.^4^, ^5^, ^6^ Recently, more and more studies have reported the use of chemoimmunotherapy in the neoadjuvant treatment of locally advanced ESCC. However, the results remain inconsistent, with major pathological response (MPR) rates of 23.5%–88.9% and pathological complete response (pCR) rates of 7.7%–51.1%. This study sought to pool these studies and analyze the efficacy and safety of neoadjuvant chemoimmunotherapy (NCIT) in clinical studies and to compare it with our real‐world experience.

## METHODS

### Clinical cohort

In this retrospective study, we identified consecutive adult patients with cT1N1‐3 M0 or cT2‐4aN0‐3 M0 ESCC who underwent NCIT followed by esophagectomy as their standard of care between January 2019 and December 2022. Eligibility criteria included: (1) adult patients with cT1N1‐3 M0 or cT2‐4aN0‐3 M0 ESCC who underwent NCIT followed by esophagectomy; (2) patients who received preoperative intravenous administration of PD‐1 inhibitor, taxane and cisplatin or carboplatin for 2–4 cycles and (3) all patients had an Eastern Cooperative Oncology Group (ECOG) performance status of 0 or 1. Patients lost to follow‐up were excluded from the study. The flow chart is shown in Figure S20. The study was approved by the Ethnical Review Committee of Fudan University Shanghai Cancer Center, and written informed consents were waived.

### Data collection and outcomes evaluation

The data were collected from the medical records of each patient including as follows: age, sex, height, weight, previous medical history, previous history of smoking and alcohol drinking, tumor stage, depth of invasion, lymph node metastasis, the type of neoadjuvant immune checkpoint inhibitor, other details of NCIT (such as therapeutic cycles), imaging outcomes, surgical data (such as surgical approach, resection margin status, postoperative complications), pathological grade, tumor regression grade (TRG), and survival follow‐up information. The TRG was assessed according to Chirieac's system.^7^, ^8^

The primary outcome was pCR (defined as the complete absence of intact tumor cells in the resected specimen and all resected lymph nodes) rate.^9^ Secondary outcomes included objective response rate (ORR), disease control rate (DCR), MPR (defined as ≤10% viable tumor cells in the resected primary tumor and all resected lymph nodes) rate, R0 resection (defined as no cancer cells at resection margins) rate, 1‐, 2‐, and 3‐years OS rates, 1‐year recurrence rate and adverse events (AEs). AEs occurred during NCIT were graded according to the National Cancer Institute Common Terminology criteria for Adverse Events (CTCAE) version 4.03.

### Literature search strategy for meta‐analysis

This meta‐analysis was performed in accordance with the Preferred Reporting Items for Systematic Review and Meta‐analysis (PRISMA) criteria (Table S1). We searched for available articles published on PubMed, Embase, Cochrane Library, Web of Science, and abstracts presented at several important international conferences before November 1, 2022, using the Medical Subject Headings (MeSH) terms shown in Table S2. To supplement the electronic search results, a manual review of the reference lists of retrieved studies was conducted to identify any relevant additional studies that might have been omitted by the electronic search. For further analysis, all the additional studies that might be of interest were retrieved. In cases where studies were serially published, only the latest and most comprehensive data was selected for assessment. We excluded studies in languages other than English and/or those involving animals. To minimize potential publication bias and duplication of results, we also excluded reviews, abstracts, case reports, conference papers, editorials and expert opinions. Additionally, we also researched some ongoing clinical studies on academic meeting and clinical trials registry platforms. As the present data were obtained from previously published studies, ethical approval and patient consent were not applicable.

### Study selection for meta‐analysis

Two coauthors (Yao Zhang, Huiting Li) conducted the screening of the title and abstracts. The full texts of all retrieved studies were assessed based on five dimensions (population, intervention, comparison, outcomes, and study design [PICOS]) in an unbiased manner. Studies that met the following criteria included: (1) population: patients with histologically confirmed advanced ESCC; (2) intervention: NCIT; (3) comparison: NCT or none; (iv) outcomes: pCR rate, MPR rate, ORR, DCR, grade 3 or higher treatment‐related AEs (TRAEs), R0 resection rate, and 1‐year recurrence rate; and (4) study design: cohort or real‐world study, or any phase clinical trial.

### Data extraction for meta‐analysis

Data were independently extracted by two coauthors (Yao Zhang, Huiting Li) from texts, tables, and figures of analyzed articles. In case of disagreements, a third author was involved in the discussion or consultation to resolve them. We collected the detailed data of each trial, including first author, year of publication, country of origin, study design, recruitment period, duration of follow‐up, number of study centers, patients' demographics and clinical information, and the incidence of AEs. In cases where articles indirectly presented survival data with Kaplan–Meier curves, GetData Graph Digitizer software was utilized to handle and extract time‐specific data. We also collected trial data (e.g., trial name, tumor type, number of patients, therapeutic strategies, pCR rate, MPR rate, R0 resection rate, ORR, DCR, clinical trial phase, and study period) from academic abstracts or ongoing prospective studies.

### Quality assessment for meta‐analysis

Two independent assessors (Jialei Wang, Hui Yu) assessed the strength of evidence for each outcome with the Grading of Recommendations, Assessment, Development and Evaluations (GRADE) system. A table summarizing the outcomes was provided to properly identify and annotate the certainty of all pooled results. Thereafter, each study was retrieved by the Newcastle‐Ottawa Scale (NOS), which accounts for selected criteria, such as selection, comparability, and outcome, to assess the quality of the study design.

### Bias assessment for meta‐analysis

To assess publication and small‐study bias, a funnel plot was generated, and then asymmetry Egger's linear regression and Begg's correlation tests were performed to inspect the suspected asymmetry for small‐study bias. To assess the risk of single‐study bias, all outcomes were analyzed with sensitivity analysis by exclusion.

### Statistical analysis

For our real‐world study, demographics, clinical data, and safety profile were tabulated using descriptive statistics. Continuous variables are expressed as median (range) or mean ± standard deviation and were compared by one‐way analysis of variance (ANOVA) or Wilcoxon sum test as appropriate. Categorical variables are presented as frequency (percentage) and were compared by *χ* ^2^‐test. *p*‐values were two‐sided, with a significance level of 0.05. The Kaplan–Meier method was used to estimate OS was estimated with the Kaplan–Meier method.

For meta‐analysis, heterogeneity across the studies was assessed by *Q*‐test and *I* ^2^ statistics. Statistical significance of heterogeneity was considered as *p* < 0.05 or *I* ^2^ > 50%. If significant heterogeneity was observed, a random‐effect model was employed; otherwise, a fixed‐effect model was utilized. The robustness of the results was checked by sensitivity analysis. Funnel plots were drawn to evaluate publication bias. Statistical analysis was conducted using R 4.0.0 (R Core Team 2020)^10^ and GraphPad Prism 6.02 (GraphPad Software Inc., San Diego, CA, USA). High‐quality figures were generated using the R packages.

## RESULTS

### Baseline characteristics of the clinical cohort

From January 2019 and February 2024, 128 eligible patients were included in this real‐world study. As of February 19, 2024, the median follow‐up duration was 29.8 months (895 days, interquartile range 20.3–36.7 m). Baseline characteristics were as expected for patients with ESCC and they were divided into four groups by TRG grade (Table 1). Their mean age was 61.98 ± 6.79 years. Most of the patients were male (89.8%), with a cT3 (93.0%), N2 (84.4%) tumor, located in the middle thoracic esophagus (71.9%). Of a total of 128 patients, 105 patients (82.0%) had stage III ESCC, whereas 22 (17.2%) experienced stage IVA disease. The immune checkpoint inhibitors used for NCIT included camrelizumab (*n* = 73, 57.0%) and pembrolizumab (*n* = 55, 43.0%).

**TABLE 1:** Patient characteristics.

	All patients (*n* = 128)	Grade 1 (*n* = 4)	Grade 2 (*n* = 67)	Grade 3 (*n* = 21)	Grade x (*n* = 36)	*p*‐value
Age (years)	61.98 ± 6.79	62.25 ± 4.11	61.39 ± 7.22	60.76 ± 5.96	63.78 ± 6.52	0.297
Sex						
Male	115 (89.8)	4 (100.0)	57 (85.1)	21 (100.0)	33 (91.7)	0.201
Female	13 (10.2)	0 (0.0)	10 (14.9)	0 (0.0)	3 (8.3)	
Height (m)	1.67 ± 0.07	1.70 ± 0.06	1.67 ± 0.07	1.68 ± 0.06	1.67 ± 0.08	0.690
Weight (kg)	63.30 ± 9.71	69.75 ± 1.12	62.45 ± 10.13	63.62 ± 9.54	64.00 ± 8.89	0.484
Body mass index (kg/m^2^)	22.59 ± 2.83	23.94 ± 2.10	22.40 ± 2.90	22.39 ± 2.84	22.90 ± 2.79	0.628
Location^a^						
2	92 (71.9)	3 (75.0)	47 (70.1)	13 (61.9)	29 (90.6)	0.477
3	36 (28.1)	1 (25.0)	20 (29.9)	8 (38.1)	7 (19.4)	
Descent stage^b^						
0	49 (38.3)	1 (25.0)	35 (52.2)	9 (42.9)	4 (11.1)	0.001
1	79 (61.7)	3 (75.0)	32 (47.8)	12 (57.1)	32 (88.9)	
Immune checkpoint inhibitor						
Camrelizumab	73 (57.0)	2 (50.0)	42 (62.7)	10 (47.6)	19 (52.8)	0.577
Pembrolizumab	55 (43.0)	2 (50.0)	25 (37.3)	11 (52.4)	17 (47.2)	
Chemotherapy						
Nab‐paclitaxel and cisplatin or carboplatin	59 (46.1)	2 (50.0)	31 (46.3)	11 (52.4)	15 (41.7)	0.960
Paclitaxel and cisplatin or carboplatin	69 (53.9)	2 (50.0)	36 (53.7)	10 (47.6)	21 (58.3)	
Cycles of chemotherapy before surgery						
Two cycles	88 (68.8)	3 (75.0)	47 (70.2)	15 (71.4)	23 (63.9)	0.940
Three cycles	22 (17.2)	1 (25.0)	10 (14.9)	3 (14.3)	8 (22.2)	
Four cycles	18 (14.0)	0 (0.0)	10 (14.9)	3 (14.3)	5 (13.9)	
cTNM classification						
II	1 (0.8)	0 (0.0)	1 (1.5)	0 (0.0)	0 (0.0)	0.476
III	105 (82.0)	2 (50.0)	55 (82.1)	16 (76.2)	32 (88.9)	
IVA	22 (17.2)	2 (50.0)	11 (16.4)	5 (23.8)	4 (11.1)	
Depth of invasion						
cT3	119 (93.0)	3 (75.0)	63 (94.0)	18 (85.7)	35 (97.2)	0.350
cT4	1 (0.8)	0 (0.0)	1 (1.5)	0 (0.0)	0 (0.0)	
cT4a	8 (6.2)	1 (25.0)	3 (4.5)	3 (14.3)	1 (2.8)	
Lymph node metastasis						
cN0	1 (0.8)	0 (0.0)	1 (1.5)	0 (0.0)	0 (0.0)	0.281
cN2	108 (84.4)	2 (50.0)	57 (85.1)	16 (76.2)	33 (91.7)	
cN3	19 (14.8)	2 (50.0)	9 (13.4)	5 (23.8)	3 (8.3)	
Clinical stage						
cStage I	42 (32.8)	0 (0.0)	14 (20.9)	2 (9.5)	26 (72.2)	<0.001
cStage II	18 (14.1)	2 (50.0)	12 (17.9)	4 (19.0)	0 (0.0)	
cStage III	57 (44.5)	1 (25.0)	37 (55.2)	11 (52.4)	8 (22.2)	
cStage IV	1 (8.6)	1 (25.0)	4 (6.0)	4 (19.0)	2 (5.6)	
Hypertension						
Yes	36 (28.1)	2 (50.0)	24 (35.8)	4 (19.0)	6 (16.7)	0.107
No	92 (71.9)	2 (50.0)	43 (64.2)	17 (81.0)	30 (83.3)	
Diabetes						
Yes	10 (7.8)	0 (0.0)	5 (7.5)	2 (9.5)	3 (8.3)	0.930
No	118 (92.2)	4 (100.0)	62 (92.5)	19 (90.5)	33 (91.7)	
Smoking						
Yes	89 (69.5)	4 (100.0)	50 (74.6)	15 (71.4)	20 (55.6)	0.115
No	39 (30.5)	0 (0.0)	17 (25.4)	6 (28.6)	16 (44.4)	
Alcohol drinking						
Yes	80 (62.5)	4 (100.0)	43 (64.2)	12 (57.1)	21 (58.3)	0.391
No	48 (37.5)	0 (0.0)	24 (35.8)	9 (42.9)	15 (41.7)	
Drug use in external hospital						
Yes	61 (47.7)	2 (50.0)	30 (44.8)	11 (52.4)	18 (50.0)	0.919
No	67 (52.3)	2 (50.0)	37 (55.2)	10 (47.6)	18 (50.0)	
Pathological grade						
0	9 (7.0)	0 (0.0)	1 (1.5)	0 (0.0)	8 (22.2)	<0.001
I	33 (25.8)	0 (0.0)	13 (19.4)	2 (9.5)	18 (50.0)	
II	17 (13.3)	2 (50.0)	11 (16.4)	4 (19.0)	0 (0.0)	
III	58 (45.3)	1 (25.0)	38 (56.7)	11 (52.4)	8 (22.2)	
IV	11 (8.6)	1 (25.0)	4 (6.0)	4 (19.0)	2 (5.6)	
Tumor regression grade						
1	32 (25.0)	0 (0.0)	0 (0.0)	0 (0.0)	32 (88.9)	<0.001
2	37 (28.9)	1 (25.0)	26 (38.8)	6 (28.6)	4 (11.0)	
3	59 (46.1)	3 (75.0)	41 (61.2)	15 (71.4)	0 (0.0)	

^a^ Location: The location of the tumor in the esophagus was determined on the basis of endoscopic findings and was divided into three segments: cervical/upper (15–25 cm from the incisor teeth), middle (25–30 cm), and lower (30–40 cm) segments of the esophagus. Location 2 and 3 represent the middle and lower segments of the esophagus.

^b^ Descent stage: Descent stage means the decrease in the stage of a patient after neoadjuvant immunochemotherapy. Zero represents that the patient's stage did not change after neoadjuvant immunochemotherapy, while 1 represents the opposite.

### Neoadjuvant therapy and response

**FIGURE 1:**
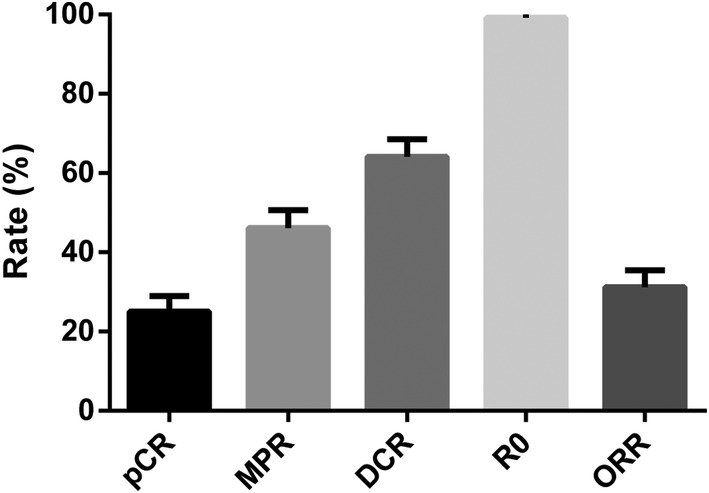
Main outcomes of neoadjuvant chemoimmunotherapy for locally advanced esophageal squamous cell carcinoma. DCR, disease control rate; MPR, major pathological response; ORR, objective response rate; pCR, pathological complete response.

A total of 88 (68.8%) patients received two cycles of NCIT, while other patients received 3–4 cycles. Regarding pathological responses, pCR was observed in 25.0% (32 of 128) of the patients, and MPR was observed in 46.1% (59 of 128) of the patients. A total of 127 patients (99.2%) achieved R0 resection. Of the 128 patients, one patient (0.8%) achieved radiological complete response, 39 patients achieved partial response (30.5%), and 42 patients (32.8%) achieved stable disease (Figure 1). DCR was observed in 64.1% (82 of 128) of the patients.

Among the 128 patients, 75 patients had postoperative PD‐L1 expression data, including 38 patients with a PD‐L1 combined positive score (CPS) of ≥10 and 37 patients with a PD‐L1 CPS of <10. In patients with PD‐L1 CPS of ≥10, the pCR and MPR rates were 2.6% (1/38) and 18.4% (7/38), respectively; in those with PD‐L1 CPS of <10, the pCR and MPR rates were 0% (0/37) and 21.6% (8/37), respectively.

### OS of ESCC based on grade

A total of 48 deaths occurred among patients with ESCC until the data cutoff date, of whom 25 were in the group of grade 2, 12 were in the group of grade 3 and nine were in the group of grade x. Median OS was 13.3 months (Figure S1). The 1‐, 2‐, and 3‐years OS rates were 91.41% (95% CI: 85.15%–95.63%), 75.00% (95% CI: 66.58%–82.23%) and 64.84% (95% CI: 55.91%–73.07%).

### Recurrence rate of ESCC

A total of 48 patients (37.5%) had recurrence until the end of follow‐up. The median recurrence interval was 11.7 months (299 days). The main recurrence sites were lymph nodes (*n* = 15, 11.72%), multisite relapse (*n* = 9, 7.0%), relapse in situ (*n* = 5, 3.91%) and lung (*n* = 5, 3.91%).

### AEs or complications

**FIGURE 2:**
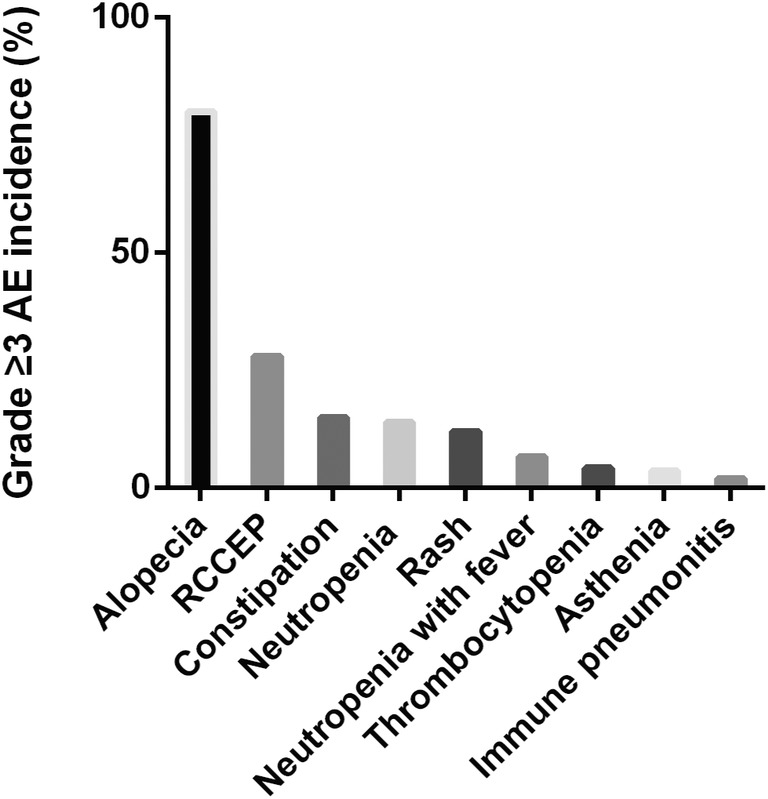
The incidence of grade 3 or higher adverse events. RCCEP, reactive cutaneous capillary endothelial proliferation.

Among the 128 patients, 123 (96.1%) patients experienced at least one TRAE. AE of ≥ grade 3 mainly included reactive cutaneous capillary endothelial proliferation (RCCEP; *n* = 36, 28.1%), neutropenia (*n* = 18, 14.1%), and rash (*n* = 15, 11.7%) (Figure 2). Surgery‐related complications mainly included pneumonia (*n* = 9, 7%), chylemia (*n* = 4, 3.1%), and wound infection (*n* = 4, 3.1%) (Table 2). No fatal AEs occurred during the follow‐up.

**TABLE 2:** Postoperative complications.

	All patients (*n* = 128)	Grade 1 (*n* = 4)	Grade 2 (*n* = 67)	Grade 3 (*n* = 21)	Grade x (*n* = 36)	*p*‐value
Pneumonia						
Yes	9 (7.0)	0 (0)	5 (7.58)	2 (9.5)	2 (5.6)	0.887
No	119 (93.0)	4 (100)	62 (92.5)	19 (90.5)	34 (94.4)	
Chylemia						
Yes	4 (3.1)	0 (0)	1 (1.5)	2 (9.5)	1 (2.8)	0.311
No	124 (96.9)	4 (100)	66 (98.5)	19 (90.5)	35 (97.2)	
Anastomic leakage						
Yes	4 (3.1)	0 (0)	1 (1.5)	2 (9.5)	1 (2.8)	0.311
No	124 (96.9)	4 (100)	66 (98.5)	19 (90.5)	35 (97.2)	
Wound infection						
Yes	4 (3.1)	0 (0)	3 (4.5)	1 (4.8)	0 (0)	0.597
No	124 (96.9)	4 (100)	64 (95.5)	20 (95.2)	36 (100)	
Second operation						
Yes	0 (0)	0 (0)	0 (0)	0 (0)	0 (0)	/
No	128 (100)	4 (100)	67 (100)	21 (100)	36 (100)	

### Literature search for meta‐analysis

As illustrated on the PRISMA flow chart (Figure S2), 226 and 14 potentially relevant articles were identified. A total of 41 articles were ineligible for inclusion following a review of the titles and abstracts. After searching for full‐text articles, 51 articles were further excluded because of duplicate publications, a lack of relevant subgroups, incomplete data, or other reasons (e.g., non‐English language). Finally, 27 articles published between 2017 and 2022,^11^, ^12^, ^13^, ^14^, ^15^, ^16^, ^17^, ^18^, ^19^, ^20^, ^21^, ^22^, ^23^, ^24^, ^25^, ^26^, ^27^, ^28^, ^29^, ^30^ were eligible for this meta‐analysis.

### Clinical outcomes in meta‐analysis

Table S3 lists the main characteristics of the studies included. All studies enrolled patients within the last 10 years, while most were published within the last 2 years.

**FIGURE 3:**
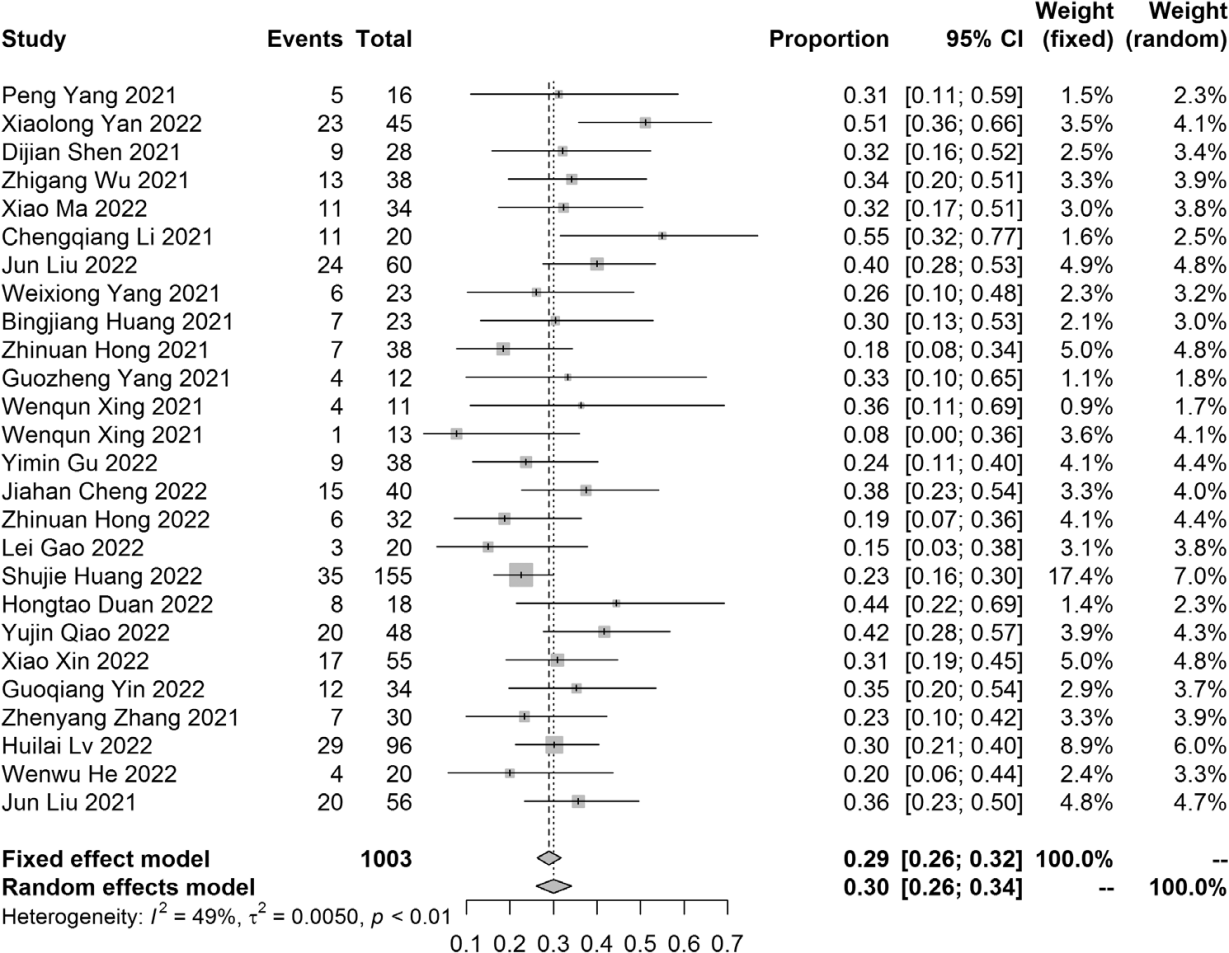
The pathological complete response rate of meta‐analysis.

**FIGURE 4:**
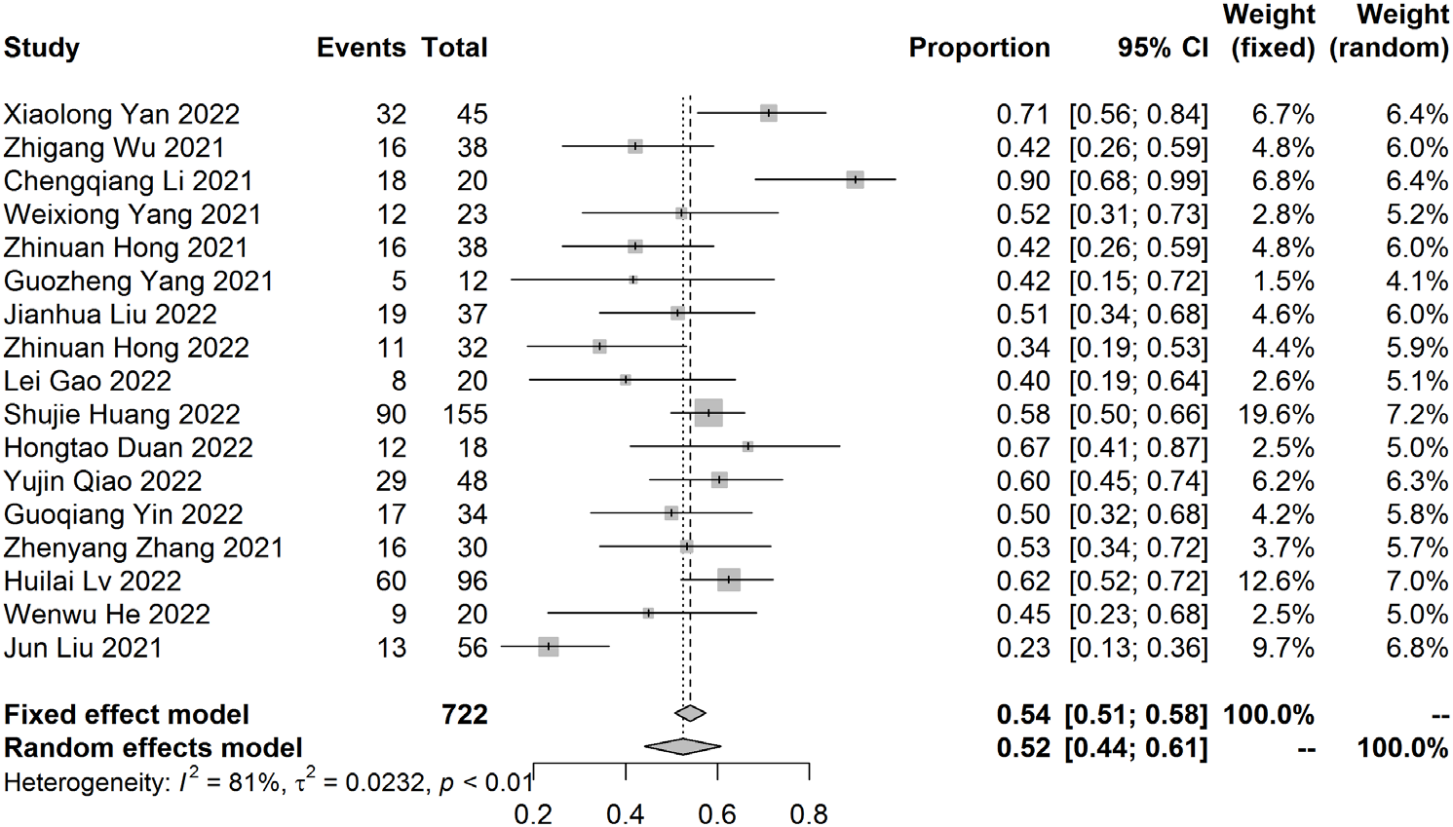
The major pathological response rate of meta‐analysis.

A total of 25 studies of NCIT provided pCR rate. The pooled pCR rate was 29% (95% CI: 26%–32%, *I* ^2^ = 49%, *p* < 0.01) (Figure 3). A total of 17 studies of NCIT provided MPR rate. The pooled MPR rate was 52% (95% CI: 44%–61%, *I* ^2^ = 81%, *p* < 0.01) (Figure 4). A total of 22 studies of NCIT provided R0 resection rate. The pooled R0 resection rate was 98% (95% CI: 97%–99%, *I* ^2^ = 31%, *P* = 0.08) (Figure S3). A total of 12 studies of NCIT provided DCR rate. The pooled DCR rate was 97% (95% CI: 95%–100%, *I* ^2^ = 56%, *p* < 0.01) (Figure S4). A total of 11 studies of NCIT provided recurrence data. The pooled 1‐year recurrence rate was 12% (95% CI: 7%–18%, *I* ^2^ = 83%, *p* < 0.01) (Figure S5).

### Safety in meta‐analysis

A total of 16 and 14 studies reported hematological and non‐hematological AEs of ≥ grade 3, respectively. The hematological AEs of ≥ grade 3 included anemia (10 studies with a pooled incidence of 3% [95% CI: 2%–5%]), leukopenia (14 studies with a pooled incidence of 12% [95% CI: 7%–17%]), neutropenia (12 studies with a pooled incidence of 7% [95% CI: 4%–11%]), thrombocytopenia (4 studies with a pooled incidence of 7% [95% CI: 3%–11%]) (Figure S6). The nonhematological grade 3 or higher AEs included fatigue (5 studies with a pooled incidence rate of 2% [95% CI: 1%–4%]), nausea or vomiting (5 studies with a pooled incidence of 3% [95% CI: 1%–5%]), pneumonia (6 studies with a pooled incidence of 8% [95% CI: 2%–13%]), rash (4 studies with a pooled incidence of 4% [95% CI: 2%–7%]) and RCCEP (3 studies with a pooled incidence of 39% [95% CI: 20%–58%]) (Figure S7).

### Publication bias and sensitivity analysis

The publication bias for the pooled proportion was evaluated with Begg's and Egger's tests (data not shown). Figures S8–S14 show the funnel plots. In general, the distribution of points in the funnel plot was more balanced, suggesting that the bias of each outcome index was slight. All sensitivity analyses related to this meta‐analysis indicated stable results (Figures S15–S19).

## DISCUSSION

The conventional therapeutic procedure for locally advanced ESCC is NCRT followed by esophagectomy, which has a pCR rate of 29%–43.2%.^31^, ^32^ Nevertheless, a higher pCR rate in NCRT did not significantly increase long‐term survival over NCT. In addition, radiotherapy may increase the difficulty of surgery and surgical complications. Neoadjuvant immunotherapy has been shown to be effective in patients with localized esophageal cancer. However, comprehensive evaluations of the effectiveness and safety of neoadjuvant immunotherapy in ESCC are rare.

Here, we provided evidence on the use of NCIT in ESCC based on our institute's experience involving 128 patients and 27 published articles including 1734 patients. In our retrospective study, the pCR rate was 25.0% (95% CI: 18.3%–33.2%). In our meta‐analysis, the pooled pCR rate was 29% (95% CI: 26%–32%), which is inferior to some other systematic reviews with a synthesized pCR of 32.5%^33^ and 32.9%.^34^ The highest pCR rate of 57.1% was from the study reported by Ma et al.,^35^ which is an abstract reporting the results and was not included in our analysis. The MPR rate for NCIT was 46.1% from our center and the pooled MPR rate was 52%, both higher than the MPR rate of 33.3% for NCT based on recent clinical trials. Moreover, the pooled R0 resection rate was 98%, and a higher R0 resection rate (99.2%) was observed in our real‐world analysis, both of which were better than the rate with NCT. These findings support the feasibility of NCIT for ESCC. According to our study, the 1‐, 2‐, and 3‐year OS rates were 91.41% (95% CI: 85.15%–95.63%), 75.00% (95% CI: 66.58%–82.23%), and 64.84% (95% CI: 55.91%–73.07%), respectively. One study conducted by Duan and his colleagues reported that the median disease‐free survival for patients with NCIT was 13.8 months among 17 patients. Most recent studies have not provided detailed survival data and long‐term follow‐up findings. Whether NCIT will provide long‐term survival benefits remain to be seen.

With regard to safety, immunotherapy did not significantly increase the incidence of TRAEs, according to the KEYNOTE‐590 trial^4^ and some other clinical trials.^36^, ^37^ In our meta‐analysis and retrospective study, although most patients experienced at least one TRAE, mainly including alopecia, RCCEP, constipation, neutropenia, and rash, these TRAEs were all manageable and did not affect the treatment. NCIT did not increase surgical difficulty in our cases, and there were no surgical complications that resulted in death. Zhang et al.^38^ found that compared to nCRT, nCIT achieved comparable pCR and MPR rates, but with significantly more lymph nodes removed during surgery, shorter operation time, reduced intraoperative blood loss, and fewer ICU admissions post‐surgery.

For patients with esophageal cancer, there are no available biomarkers to precisely predict the therapeutic outcomes of immunotherapy. The most commonly employed biomarker is PD‐L1 expression. However, it is still debatable if PD‐L1 expression levels are associated with pathological response in patients treated with neoadjuvant immunotherapy. Several clinical trials^39^, ^40^ suggested that patients could benefit from immunotherapy, irrespective of PD‐L1 expression levels. Some other studies indicated that the pCR group showed considerably higher level of PD‐L1 before treatment when compared to the non‐pCR group 28. In our center, according to the post‐surgery biopsy, 38 patients were PD‐L1 positive with CPS ≥10, and their pCR and MPR rates were 2.6% (1/38) and 18.4% (7/38), respectively. A total of 37 patients were PD‐L1 negative with CPS <10, and their pCR and MPR rates were 0% (0/37) and 21.6% (8/37), respectively. No significant differences were found between the two groups in our study. Since the baseline PD‐L1 expression level was not evaluated, the association between it and pathological response could not be evaluated in our real‐world study. We were also unable to run a meta‐regression analysis to include these putative biomarkers due to the lack of research data.

There were several limitations in the present study. First, the disease stage could be a factor for the disparities between the MPR rate and R0 resection rate. However, in our study, we were unable to measure the influence of disease stage on the clinical outcomes. Second, different immune checkpoint inhibitors were involved in our study, which could make the current study's conclusions less generalizable. Third, the most studies involved in our meta‐analysis were single‐arm studies, which may lead to the instability and bias of the findings. Large randomized controlled trials are still warranted in the future.

In conclusion, NCIT has an acceptable safety profile, obtained pathological downstaging, and may bring survival benefits for locally advanced esophageal squamous cell carcinoma, indicating its application value in resectable esophageal squamous cell carcinoma.

## AUTHOR CONTRIBUTIONS

Study concept and design: Hui Yu, Yang Zhang, Bin Li, Yawei Zhang and Jiaqing Xiang. Acquisition, analysis, or interpretation of data: Yao Zhang, Huiting Li and Hui Yu. Patients follow‐up and study supervision: All authors. Statistical analysis: Yao Zhang and Huiting Li. Drafting of the manuscript: Yao Zhang and Huiting Li. All authors contributed to manuscript revision, read, and approved the submitted version.

## CONFLICT OF INTEREST STATEMENT

The authors declare that the research was conducted in the absence of any commercial or financial relationships that could be construed as a potential conflict of interest.

## Supporting information


**Data S1.** Supporting Information.

## Data Availability

The raw data supporting the conclusions of this article will be made available by the authors without undue reservation.
