# Supplementary material for: Neoadjuvant chemoimmunotherapy for locally advanced esophageal squamous cell carcinoma: Data from literature review and a real‐world analysis
Source: Thorac Cancer. 2024 Mar 26;15(13):1072–81. doi: 10.1111/1759-7714.15291 (PMC11062853; doi:10.1111/1759-7714.15291)
Supplement: Supplementary file 1 — Data S1. Supporting Information. [file TCA-15-1072-s001.docx]

Supplementary Material

# Supplementary Figures


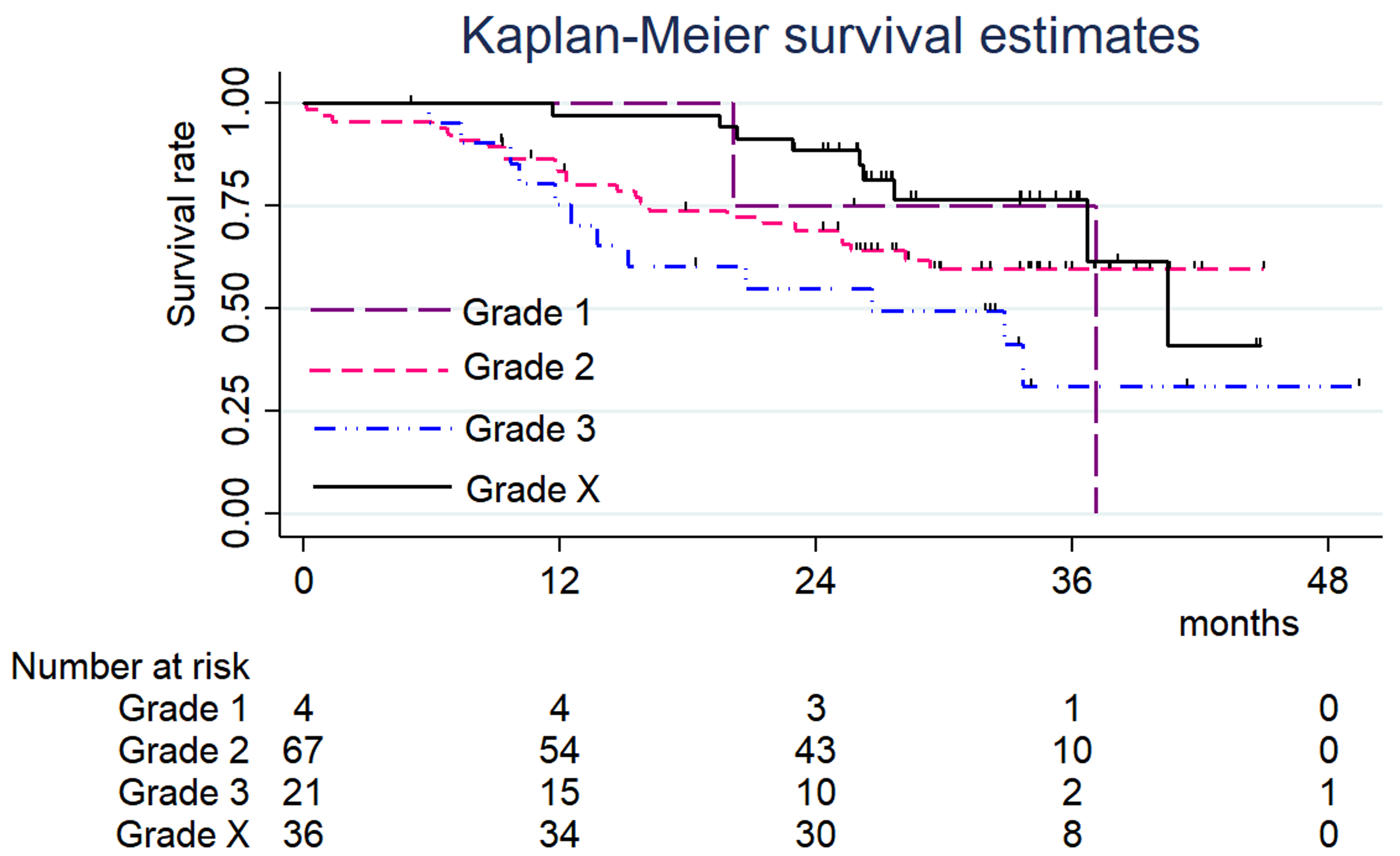


**Supplementary Figure 1.** The Kaplan-Meier plot for overall survival in patients with esophageal squamous cell carcinoma.


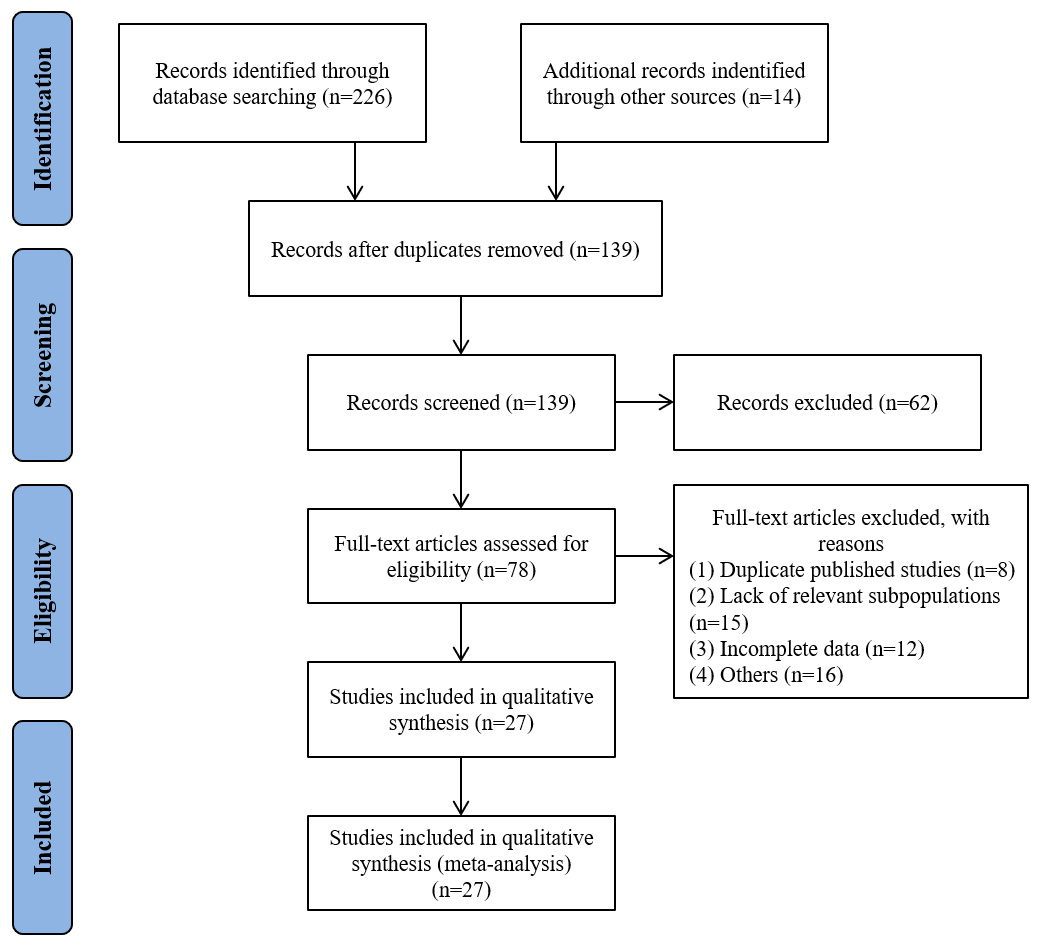
**Supplementary Figure 2.** Preferred Reporting Items for Systematic Reviews and Meta-Analyses (PRISMA) flow chart of meta-analysis.


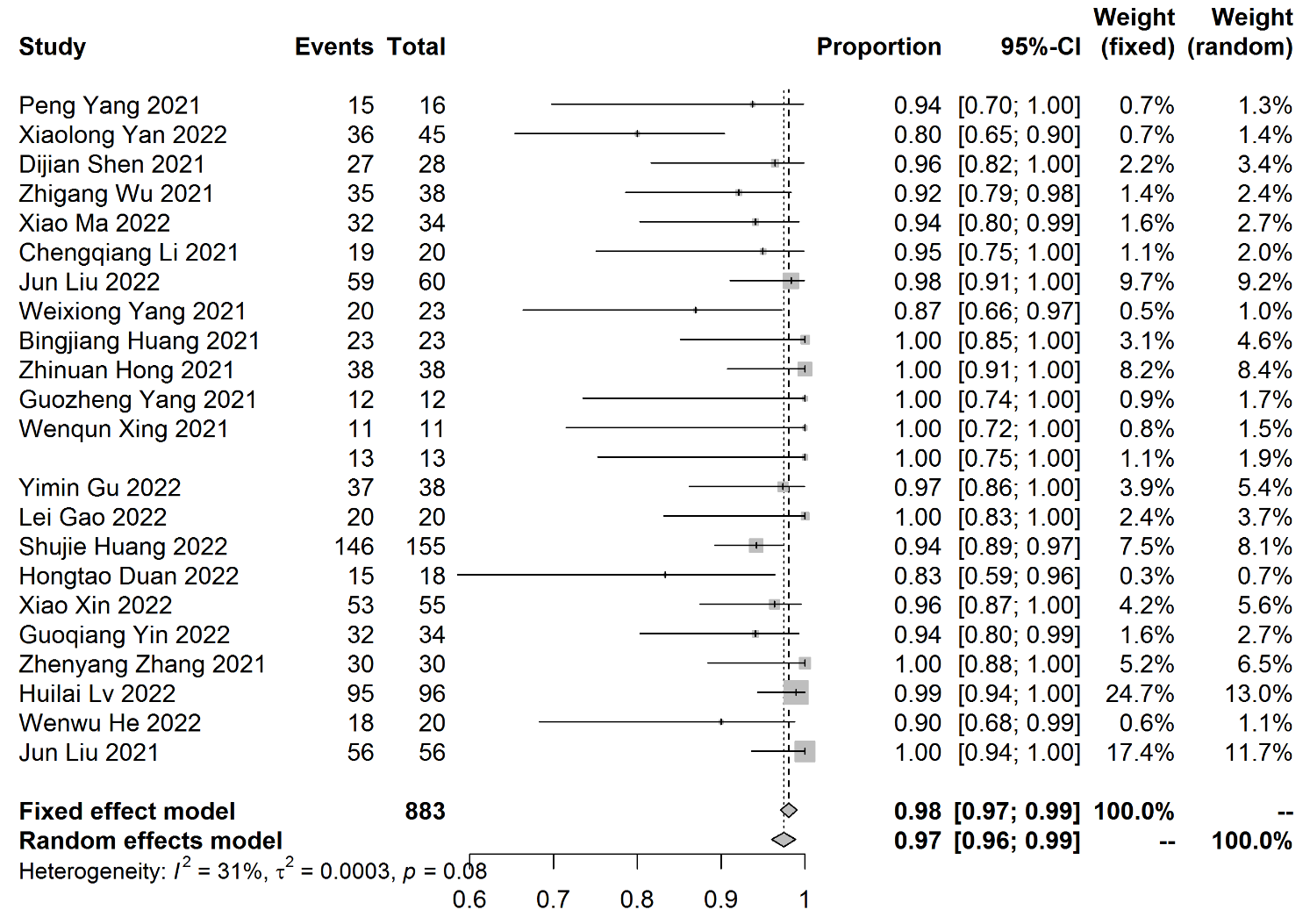


**Supplementary Figure 3.** The R0 resection rate of meta-analysis


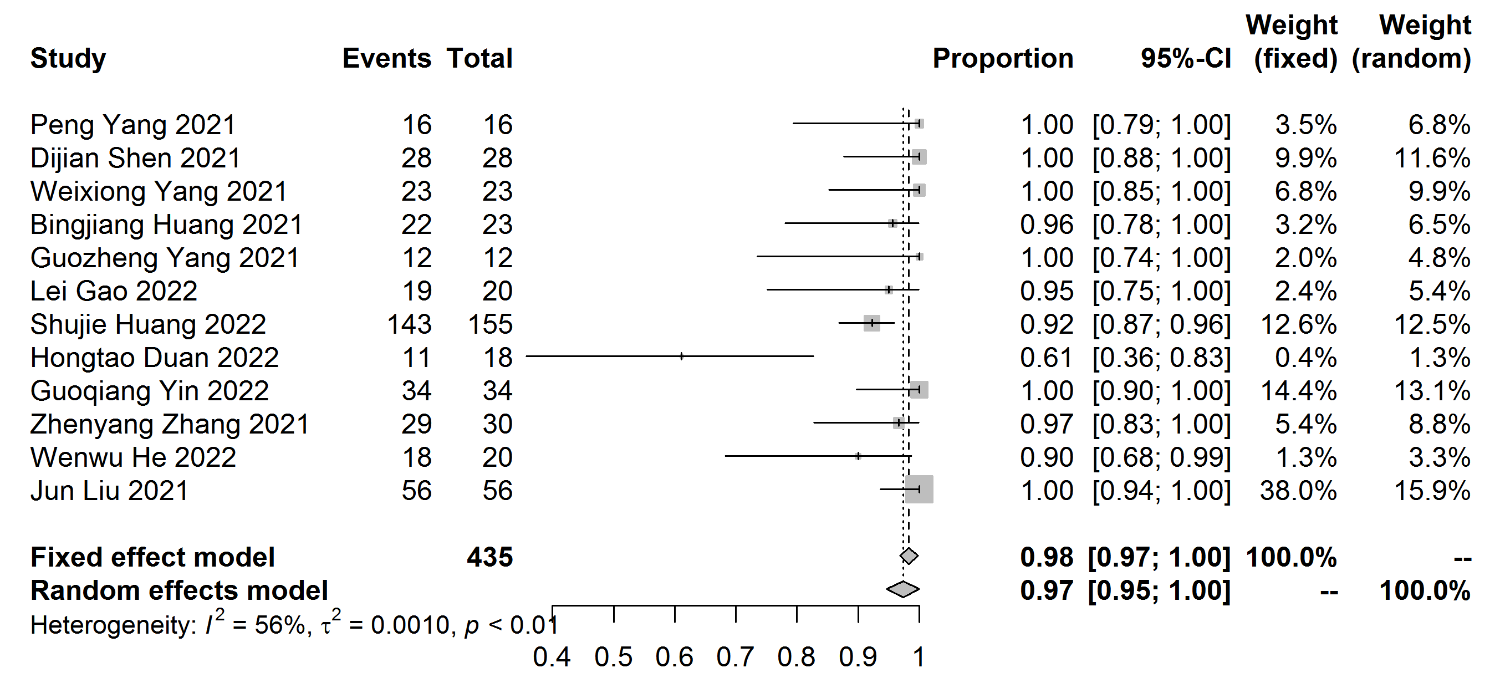


**Supplementary Figure 4.** The disease control rate of meta-analysis


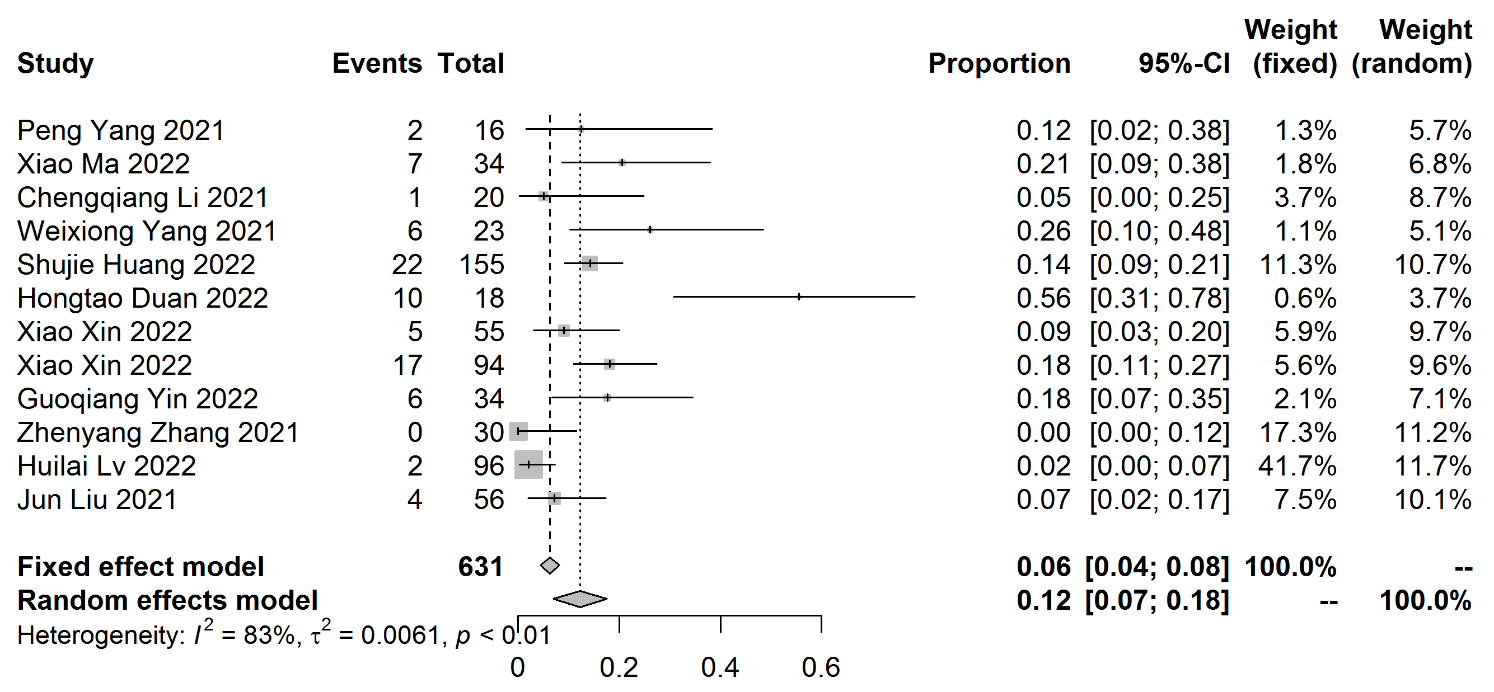


**Supplementary Figure 5.** The recurrence rate of meta-analysis.


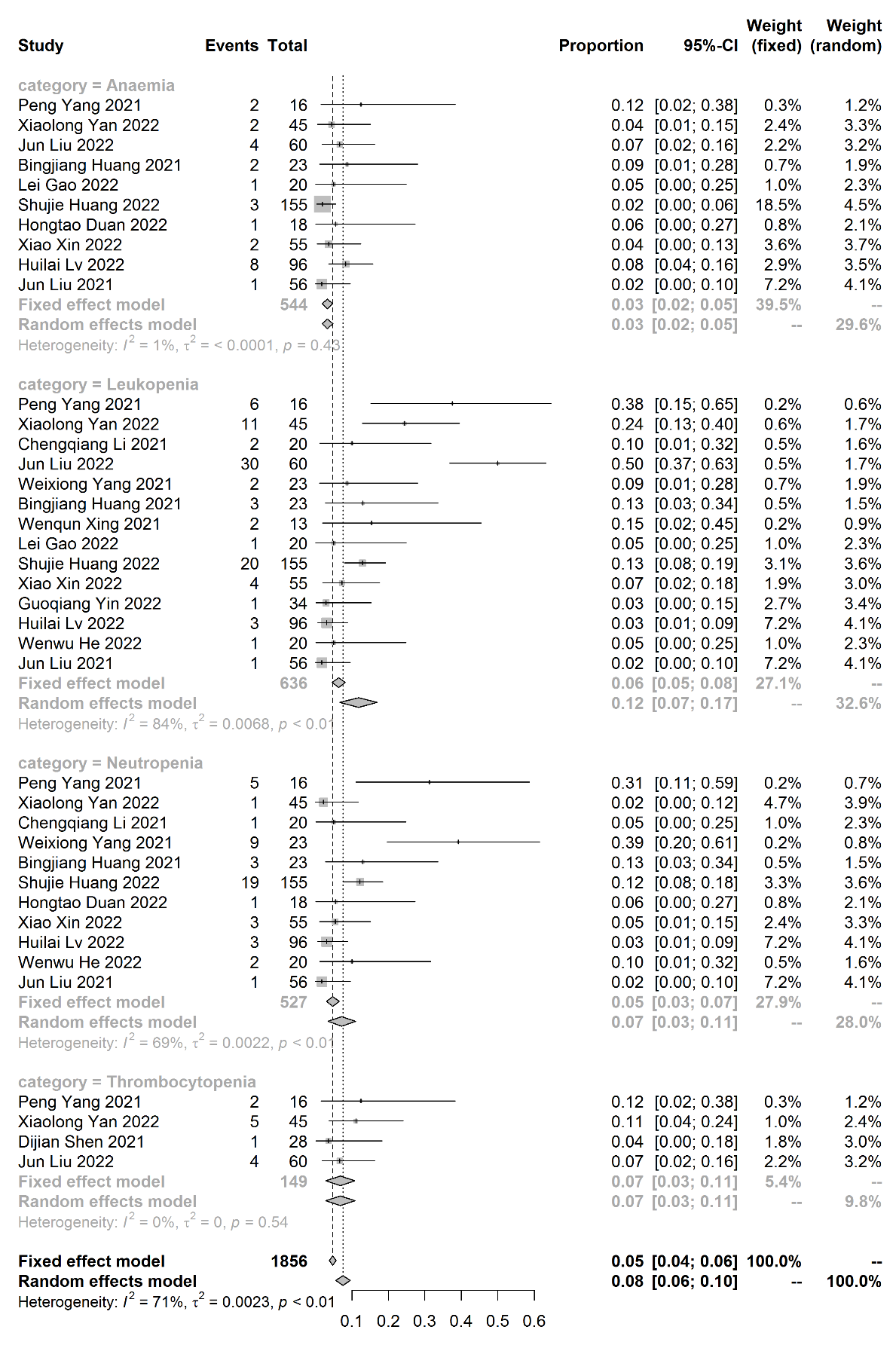


**Supplementary Figure 6.** The incidence of hematologic grade 3 or higher adverse events of meta-analysis


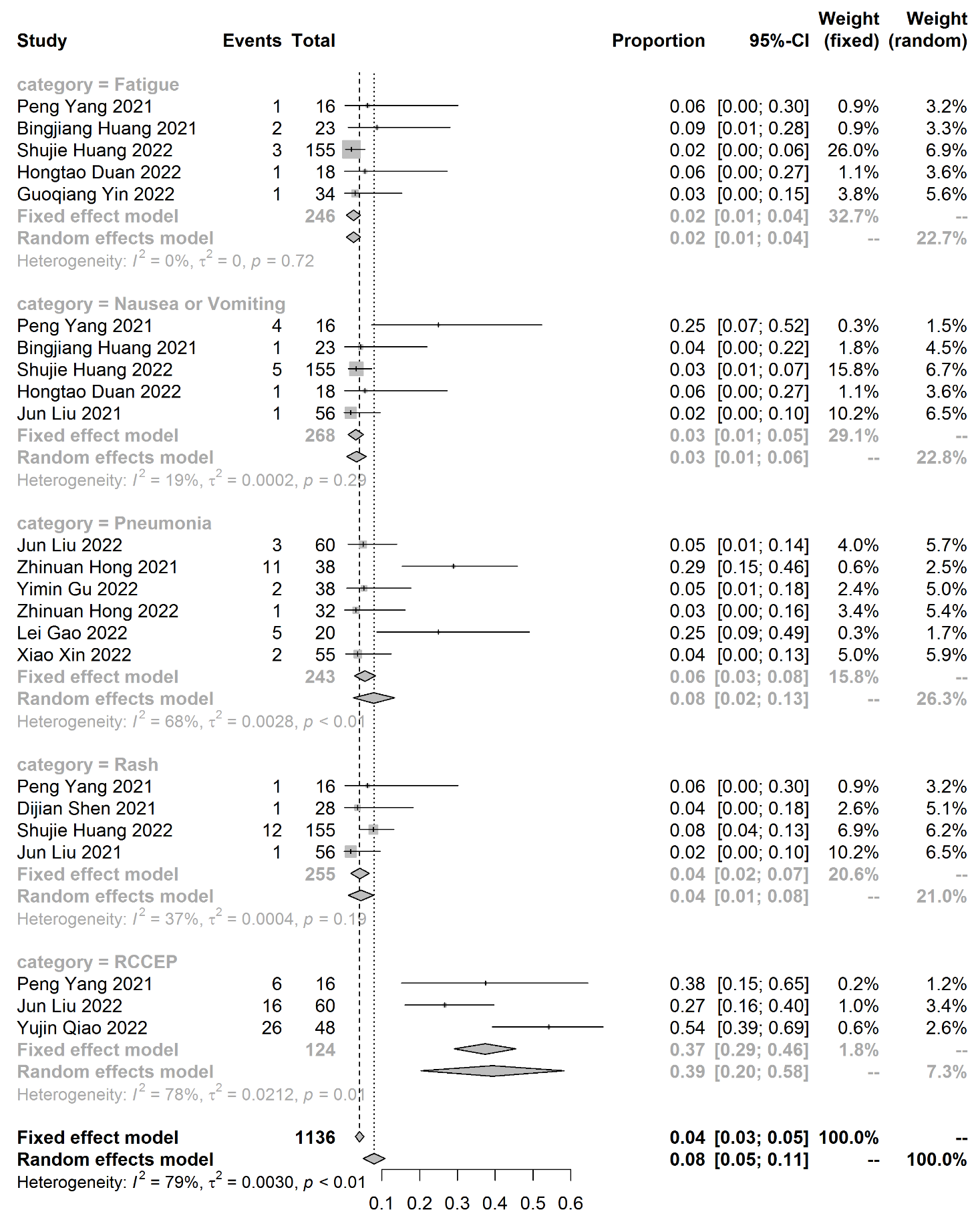


**Supplementary Figure 7.** The incidence of non-hematologic grade 3 or higher adverse events of meta-analysis. RCCEP, reactive cutaneous capillary endothelial proliferation.


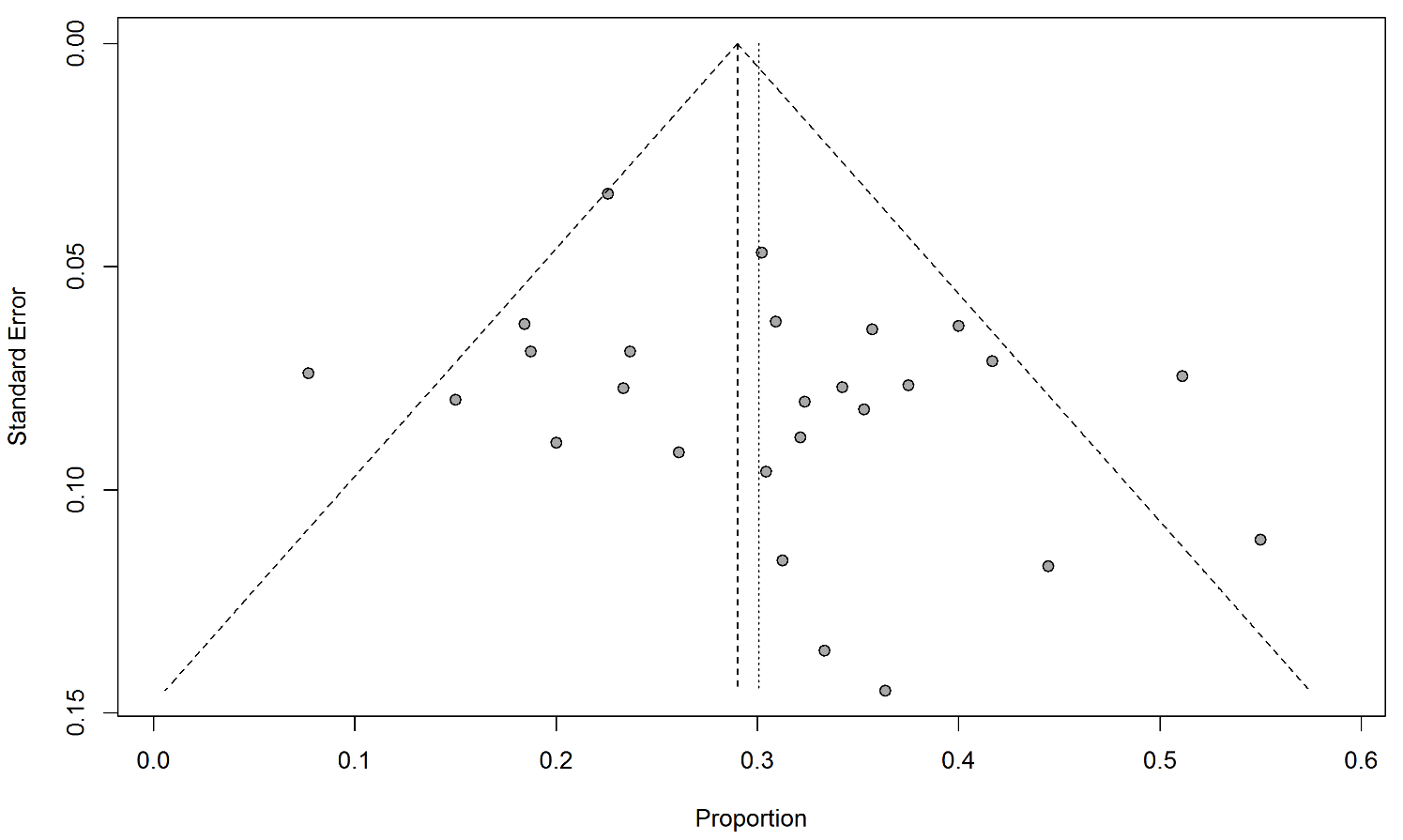


**Supplementary Figure 8.** The funnel plots of meta-analysis for pathological complete response rate.


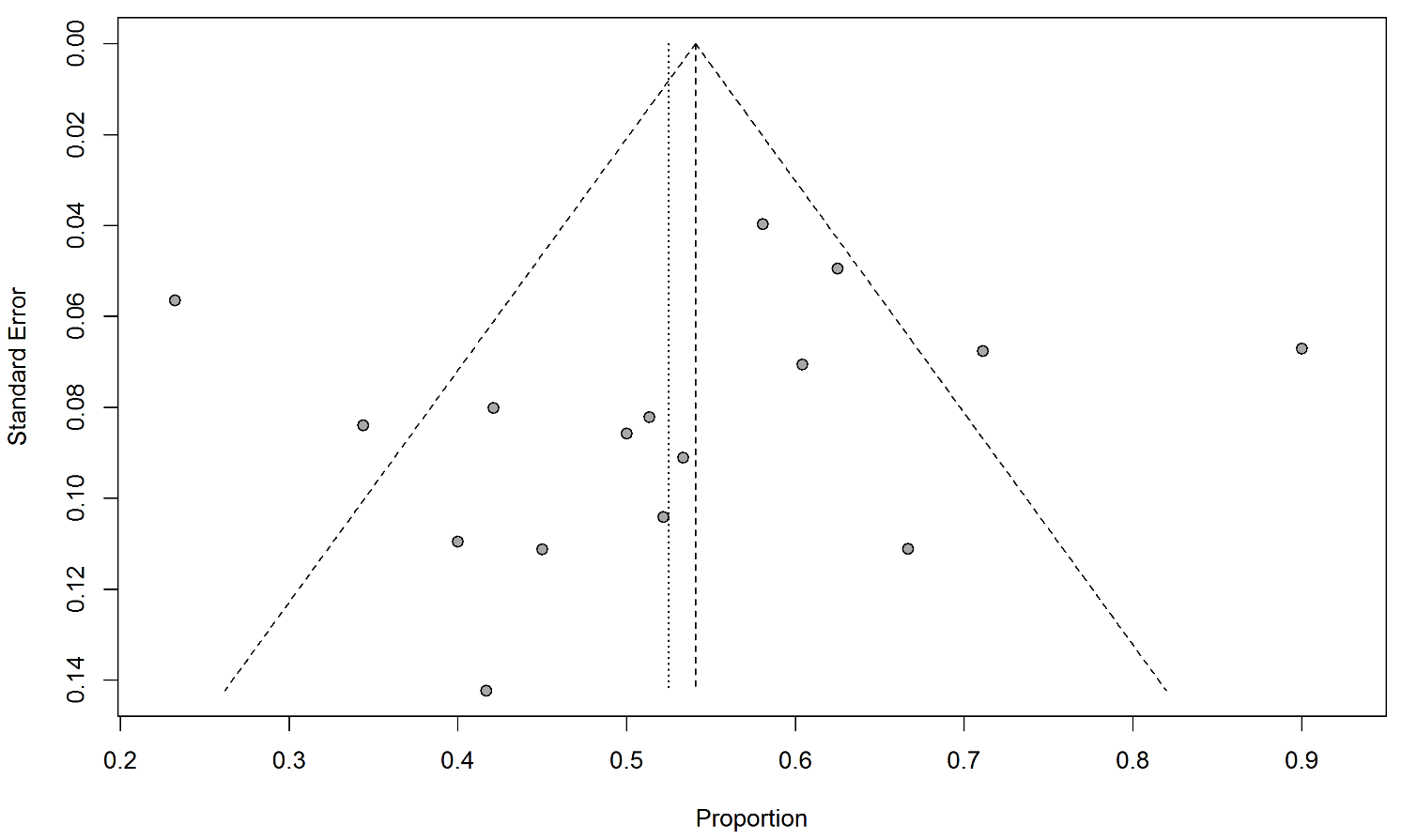


**Supplementary Figure 9.** The funnel plots of meta-analysis for major pathological response rate.


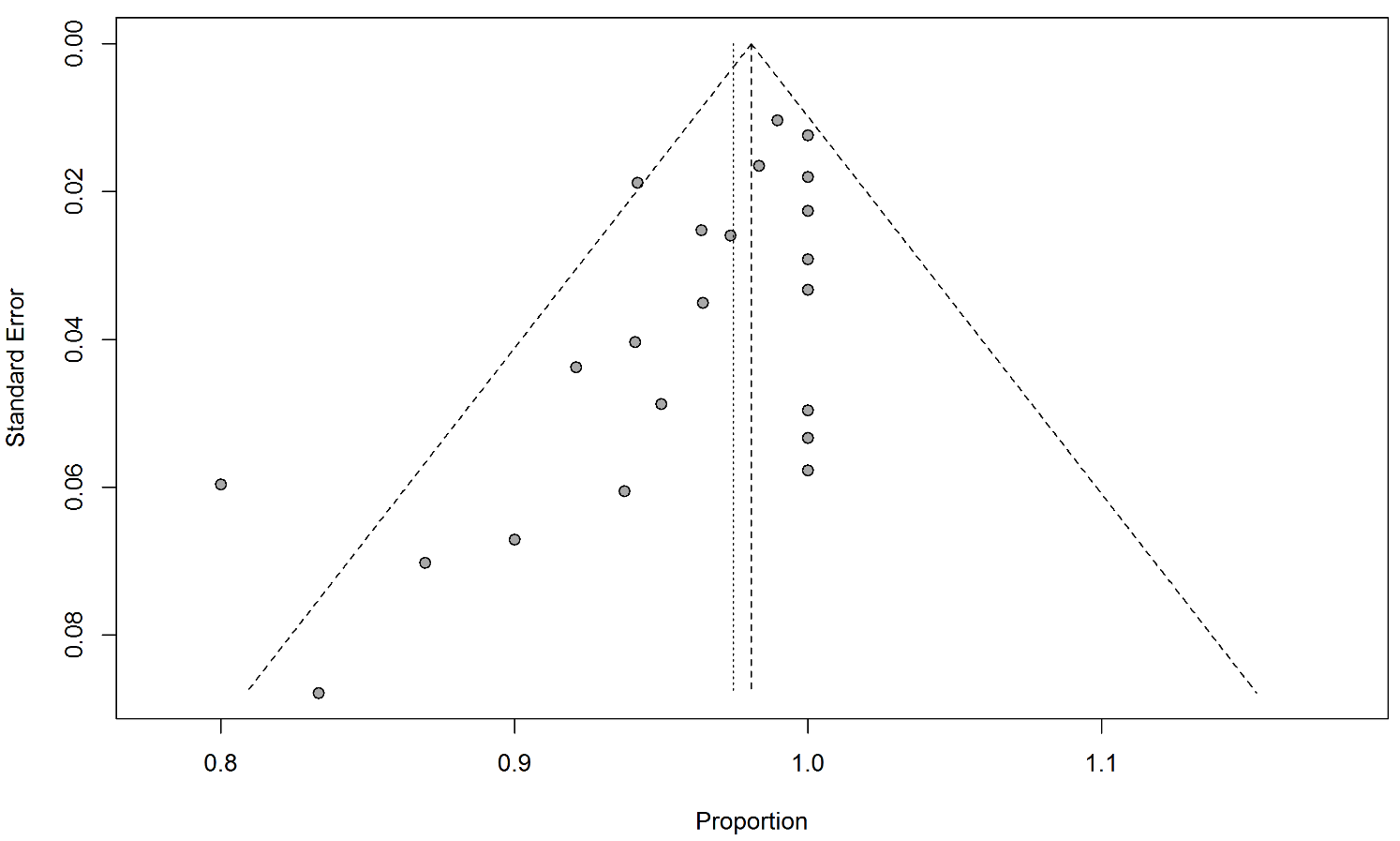


**Supplementary Figure 10.** The funnel plots of meta-analysis for R0 resection rate.


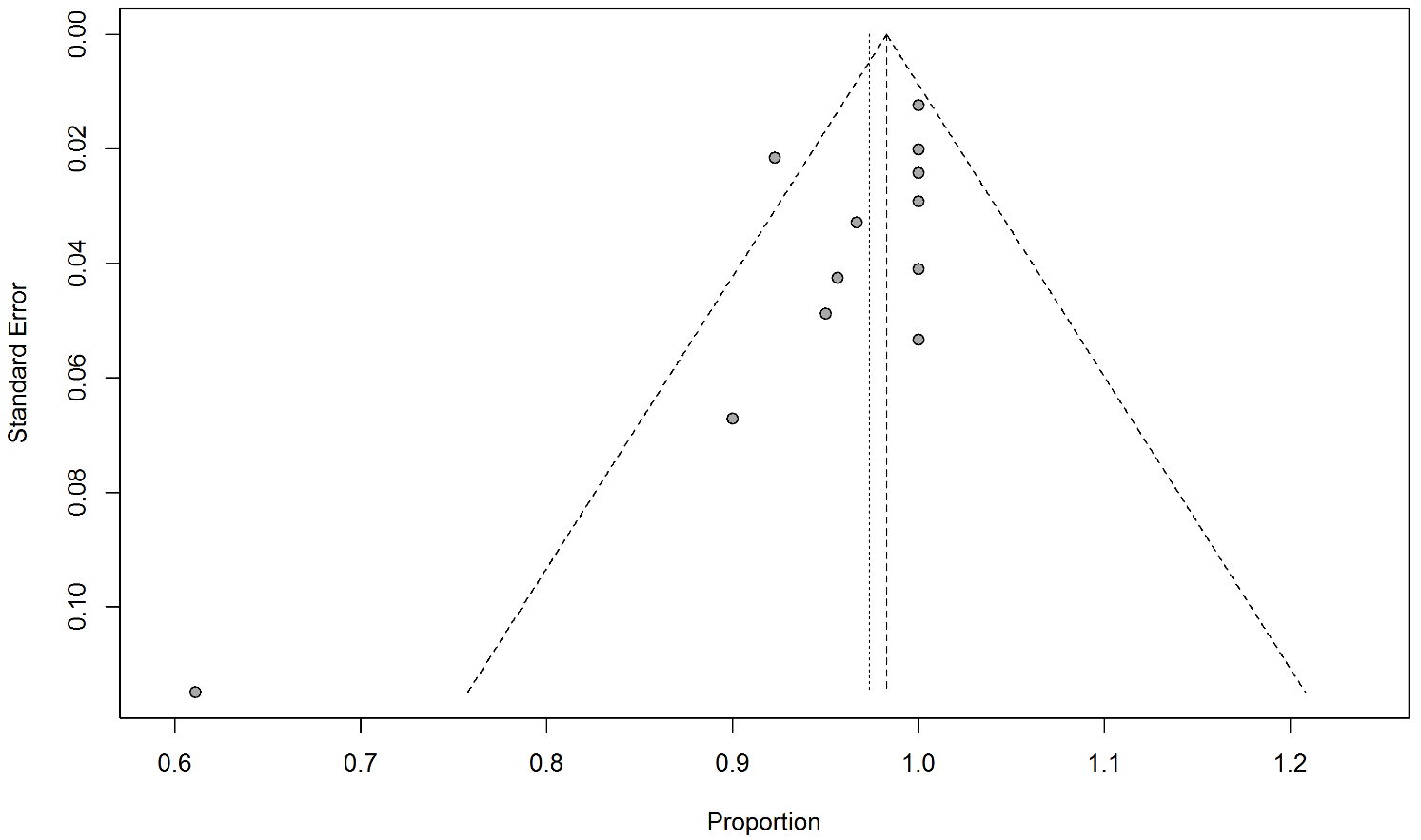


**Supplementary Figure 11.** The funnel plots of meta-analysis for disease control rate.


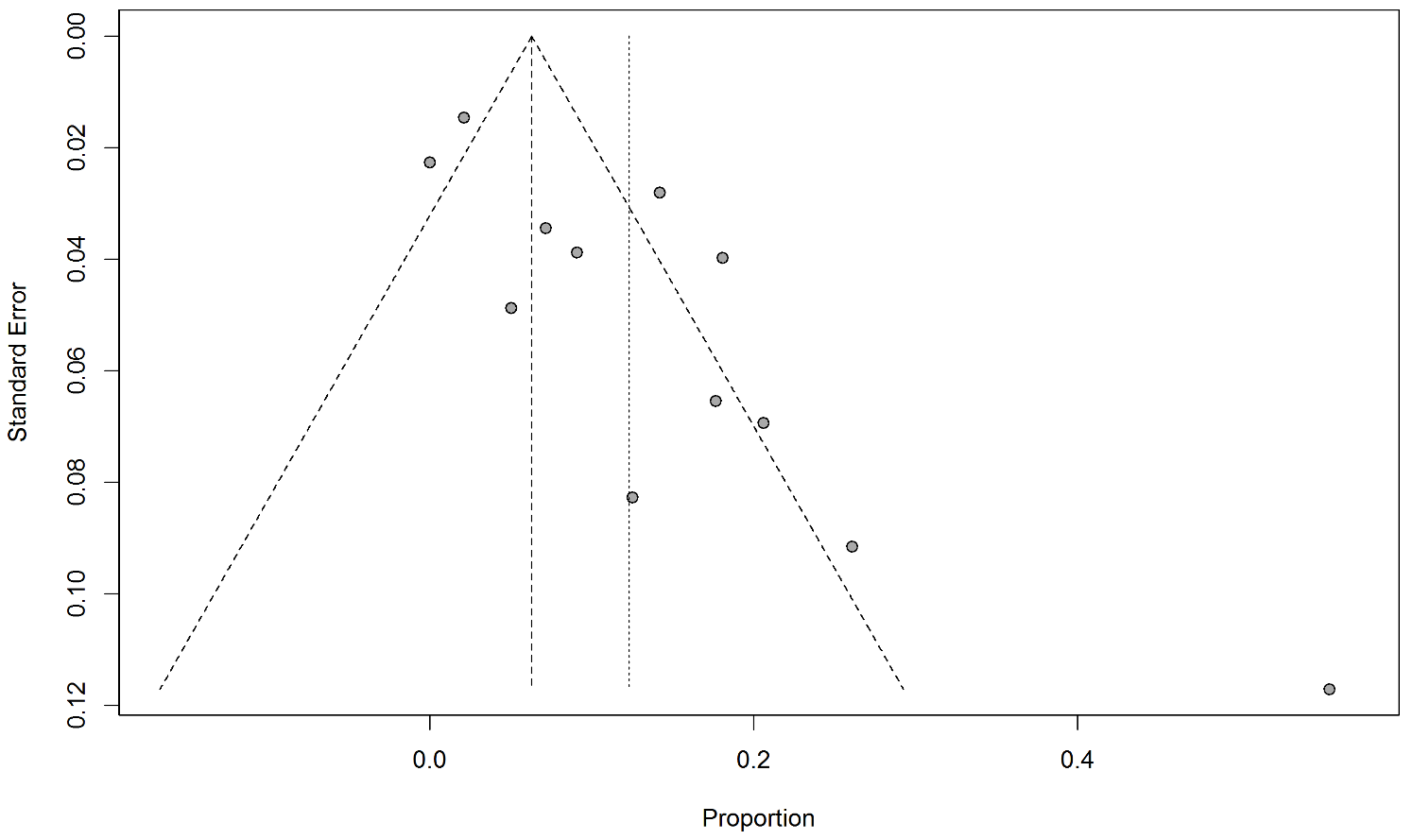


**Supplementary Figure 12.** The funnel plots of meta-analysis for recurrence rate.


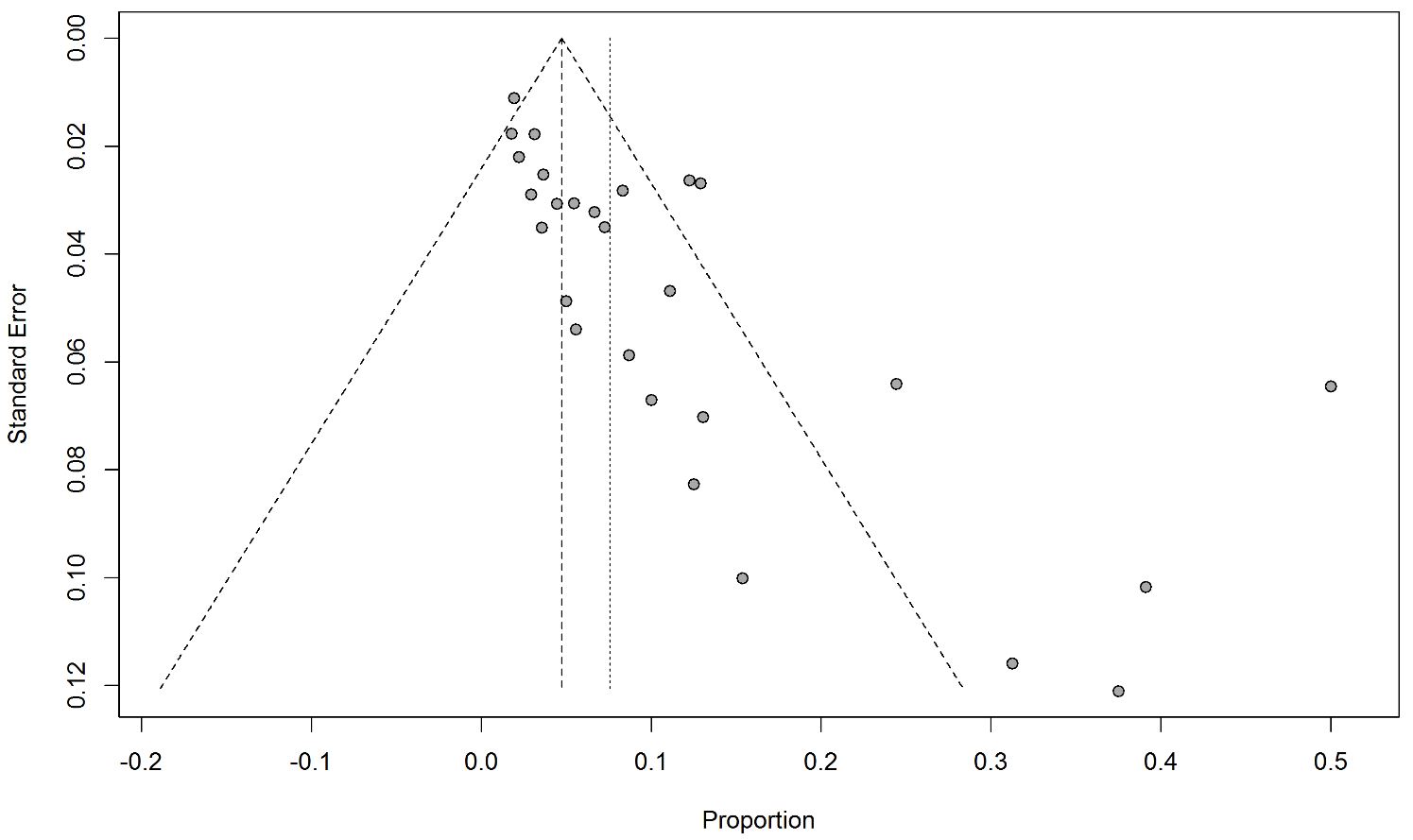


**Supplementary Figure 13.** The funnel plots of meta-analysis for the incidence of hematologic grade 3 or higher adverse events.


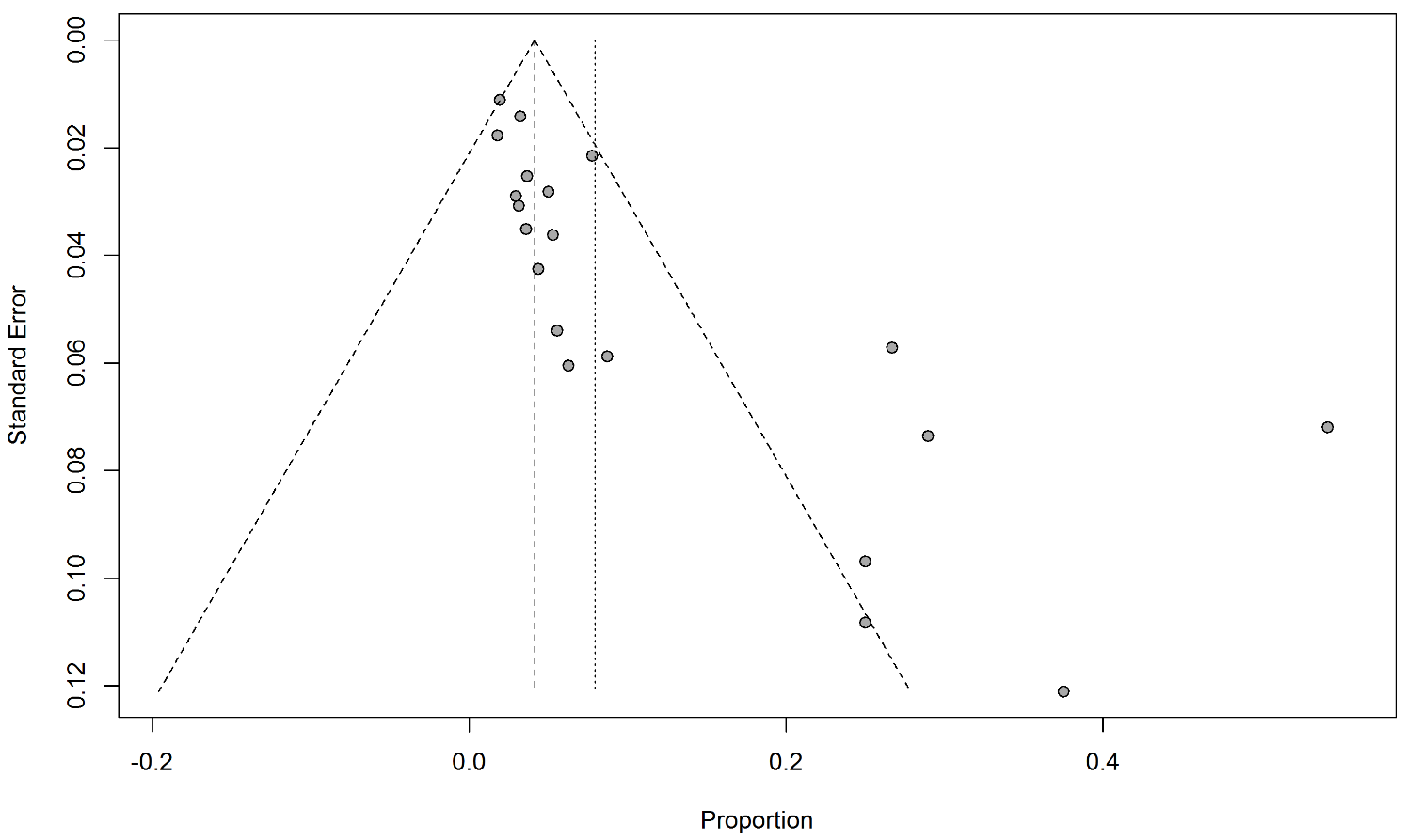


**Supplementary Figure 14.** The funnel plots of meta-analysis for the incidence of non-hematologic grade 3 or higher adverse events.


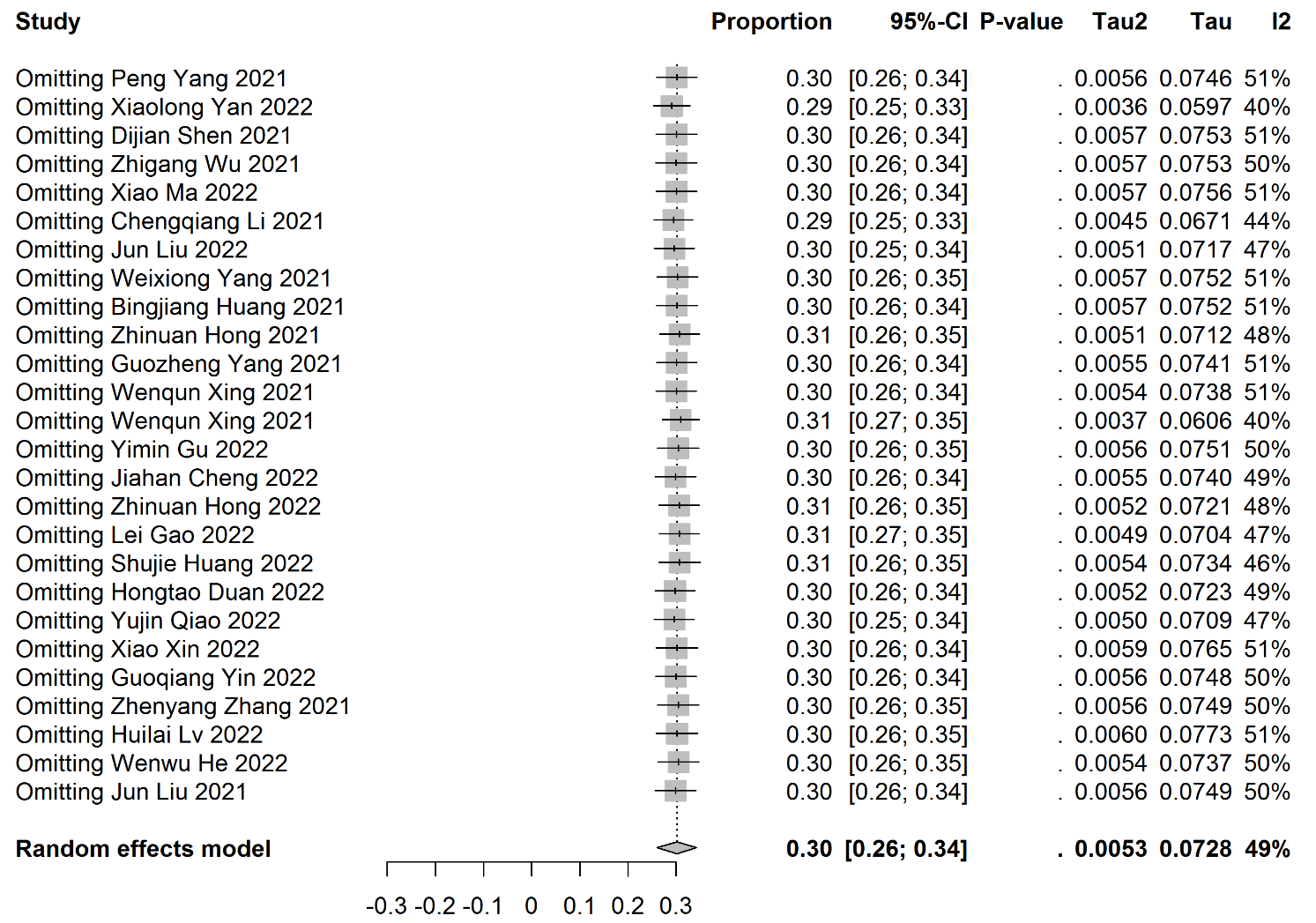


**Supplementary Figure 15.** The sensitivity analysis plot of meta-analysis for pathological complete response rate.


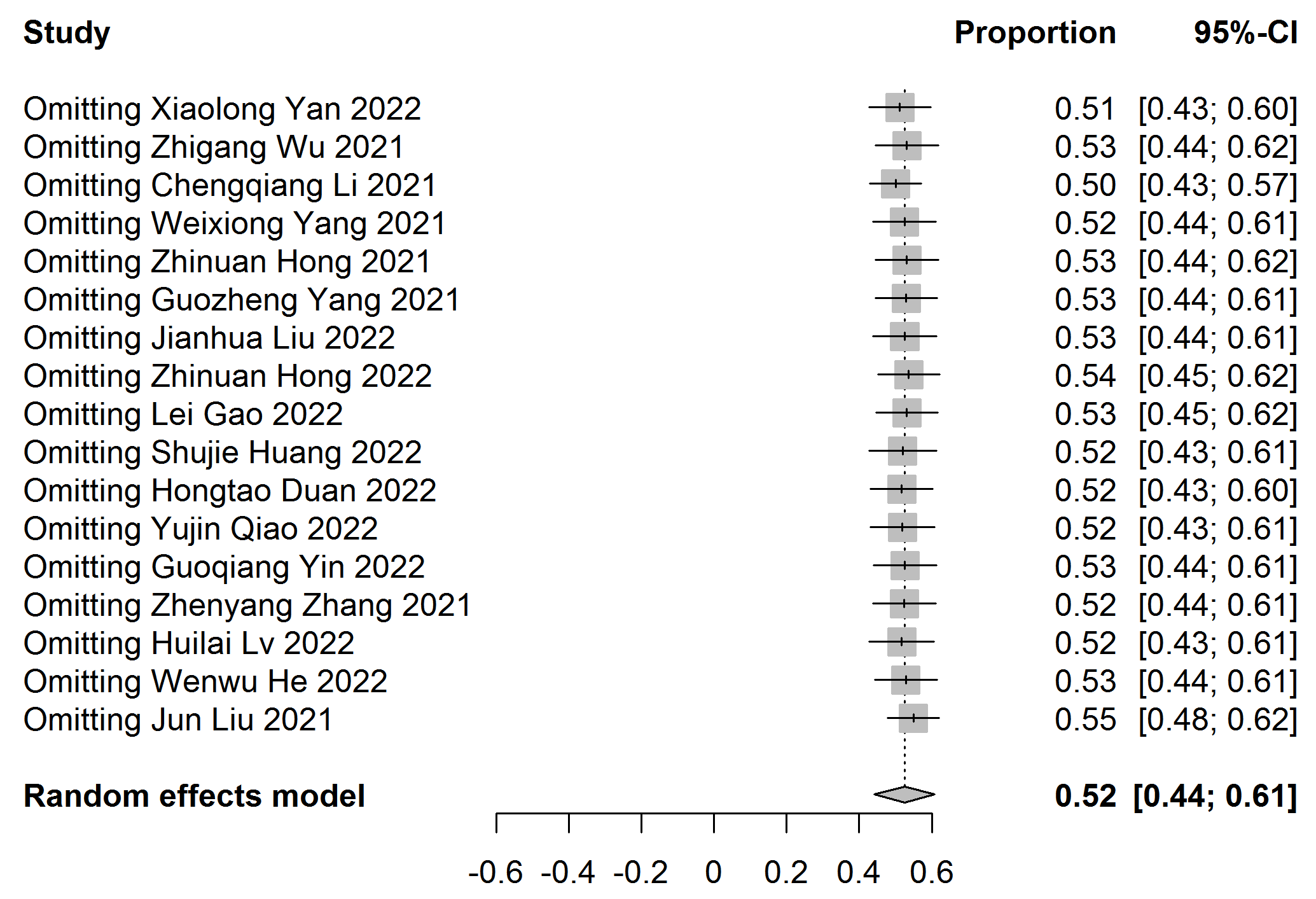


**Supplementary Figure 16.** The sensitivity analysis plot of meta-analysis for major pathological response rate.


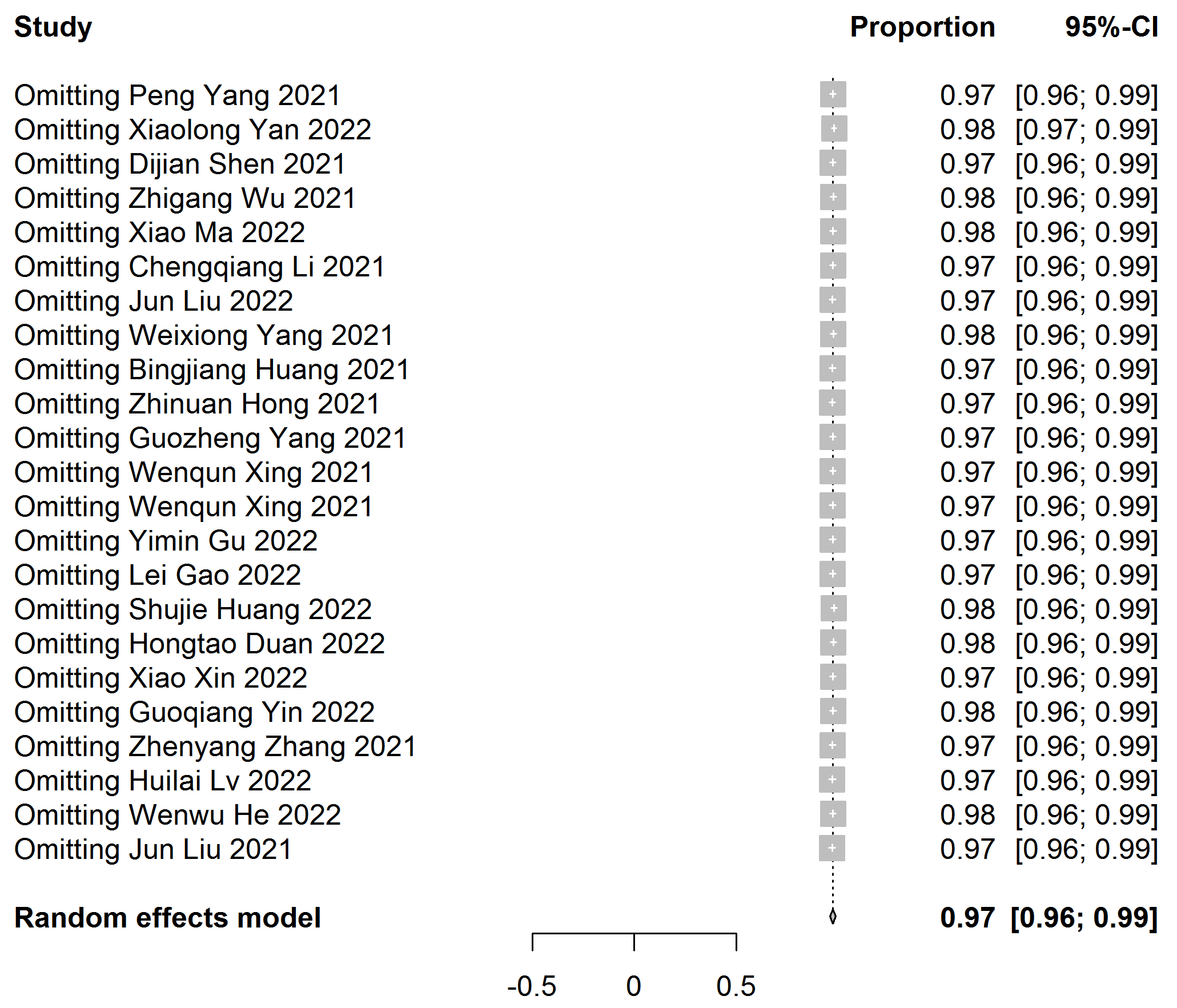


**Supplementary Figure 17.** The sensitivity analysis plot of meta-analysis for R0 resection rate.


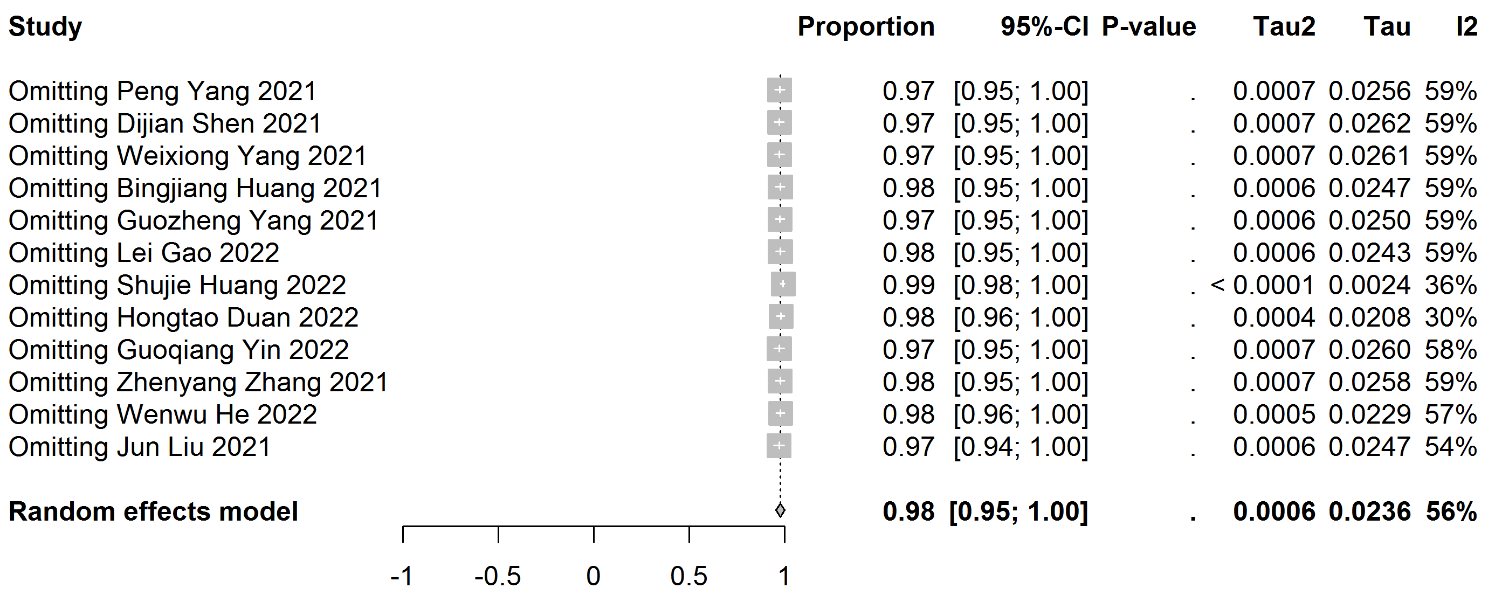


**Supplementary Figure 18.** The sensitivity analysis plot of meta-analysis for disease control rate.


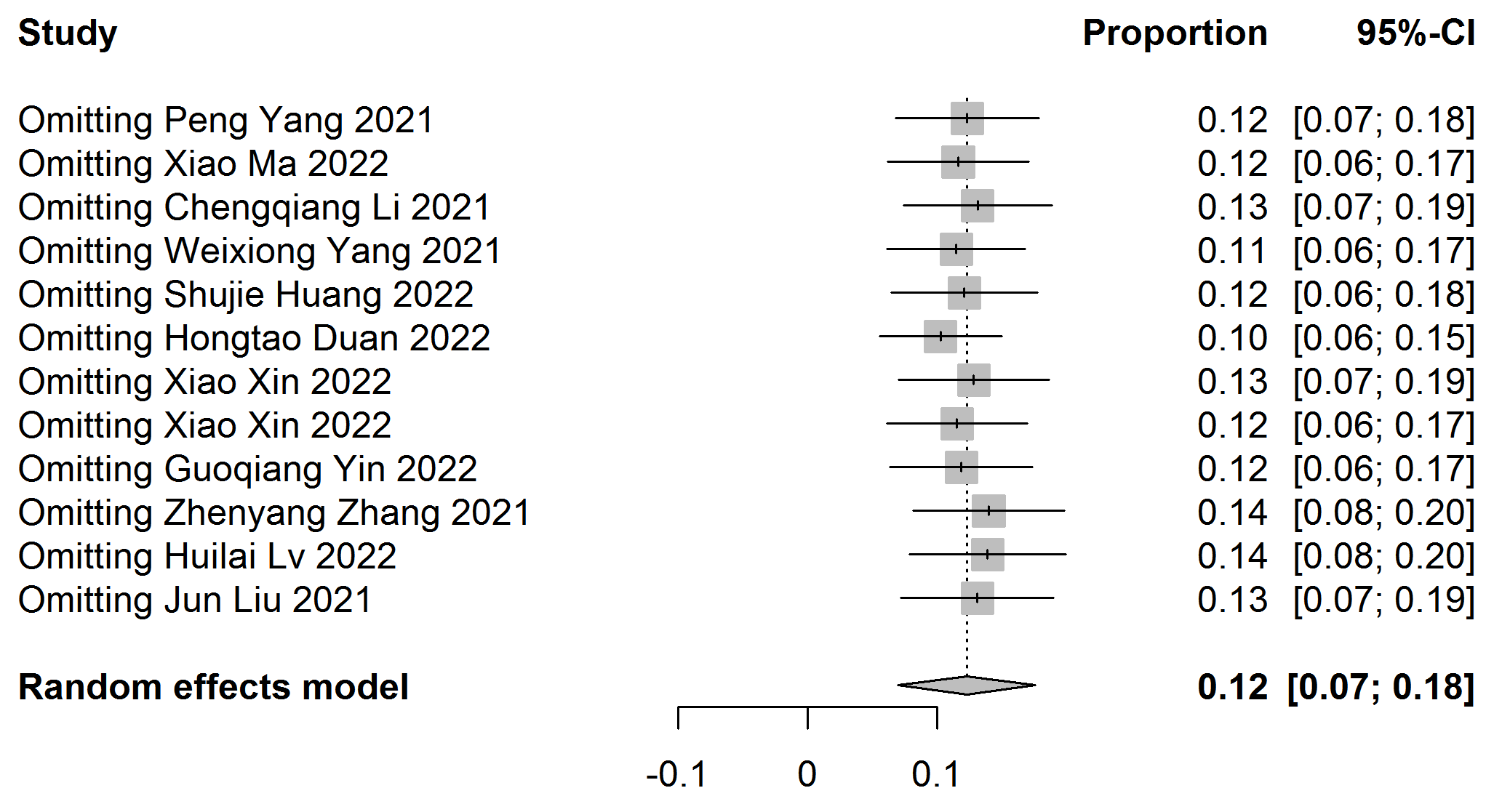
**Supplementary Figure 19.** The sensitivity analysis plot of meta-analysis for recurrence rate.


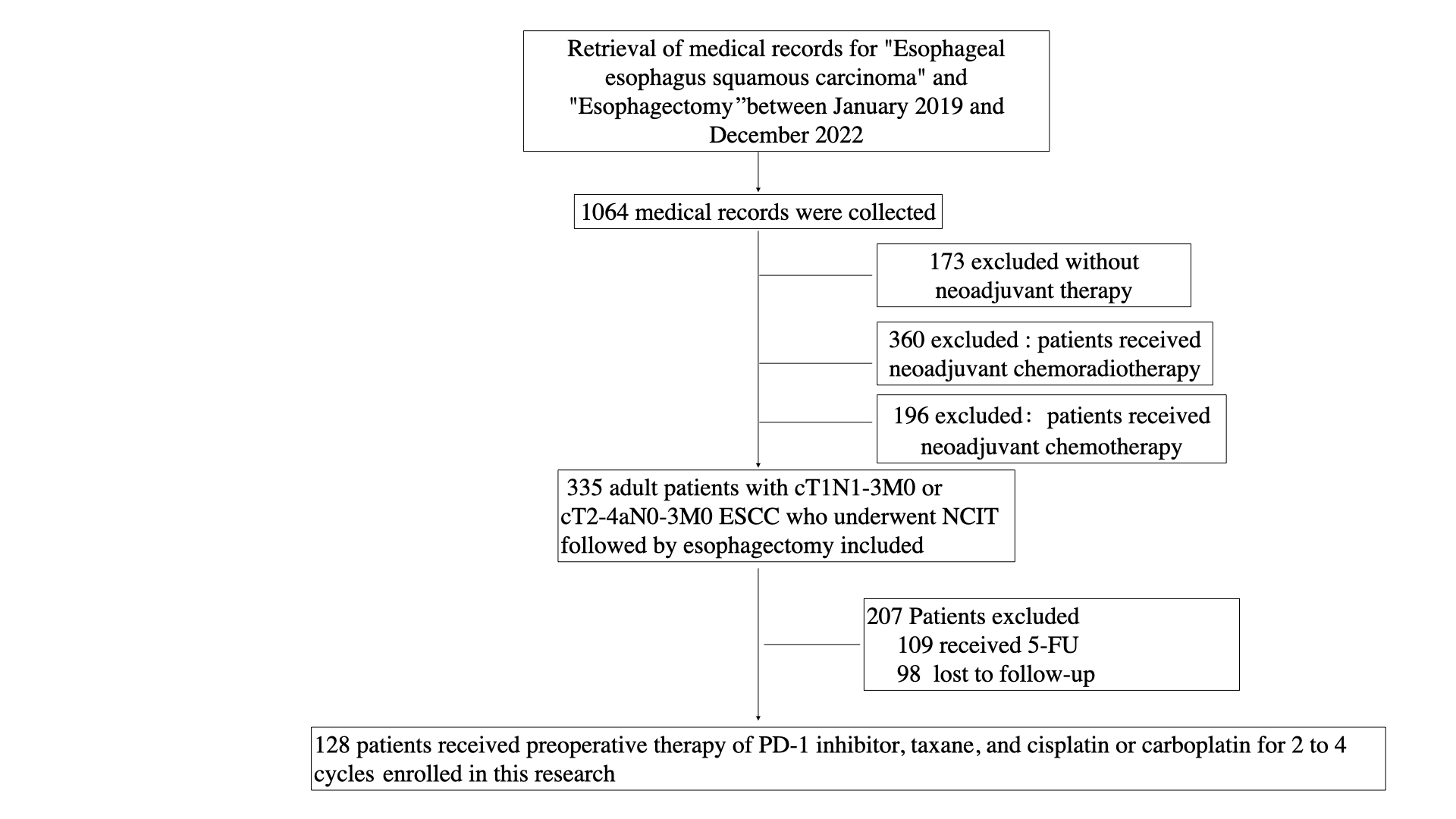
**Supplementary Figure 20.** The flow chart of patient selection.

# Supplementary Tables

**Supplementary Table 1.** Preferred Reporting Items for Systematic Reviews and Meta-Analyses (PRISMA) Checklist

| **Section/Topic** | **Item #** | **Checklist Item** | **Reported on Page #** |
| --- | --- | --- | --- |
| **TITLE** |  |  |  |
| Title | 1 | Identify the report as a systematic review incorporating a meta-analysis (or related form of meta-analysis). |  |
| **ABSTRACT** |  |  |  |
| Structured summary | 2 | Provide a structured summary including, as applicable:  **Background:** main objectives  **Methods:** data sources; study eligibility criteria, participants, and interventions; study appraisal; and synthesis methods, such as network meta-analysis.  **Results:** number of studies and participants identified; summary estimates with corresponding confidence/credible intervals; treatment rankings may also be discussed. Authors may choose to summarize pairwise comparisons against a chosen treatment included in their analyses for brevity.  **Discussion/Conclusions:** limitations; conclusions and implications of findings.  **Other:** primary source of funding; systematic review registration number with registry name. |  |
| **INTRODUCTION** |  |  |  |
| Rationale | 3 | Describe the rationale for the review in the context of what is already known, including mention of why a network meta-analysis has been conducted. |  |
| Objectives | 4 | Provide an explicit statement of questions being addressed, with reference to participants, interventions, comparisons, outcomes, and study design (PICOS). |  |
| **METHODS** |  |  |  |
| Protocol and registration | 5 | Indicate whether a review protocol exists and if and where it can be accessed (e.g., Web address); and, if available, provide registration information, including registration number. |  |
| Eligibility criteria | 6 | Specify study characteristics (e.g., PICOS, length of follow-up) and report characteristics (e.g., years considered, language, publication status) used as criteria for eligibility, giving rationale. Clearly describe eligible treatments included in the treatment network, and note whether any have been clustered or merged into the same node (with justification). |  |
| Information sources | 7 | Describe all information sources (e.g., databases with dates of coverage, contact with study authors to identify additional studies) in the search and date last searched. |  |
| Search | 8 | Present full electronic search strategy for at least one database, including any limits used, such that it could be repeated. |  |
| Study selection | 9 | State the process for selecting studies (i.e., screening, eligibility, included in systematic review, and, if applicable, included in the meta-analysis). |  |
| Data collection process | 10 | Describe method of data extraction from reports (e.g., piloted forms, independently, in duplicate) and any processes for obtaining and confirming data from investigators. |  |
| Data items | 11 | List and define all variables for which data were sought (e.g., PICOS, funding sources) and any assumptions and simplifications made. |  |
| **Geometry of the network** | **S1** | Describe methods used to explore the geometry of the treatment network under study and potential biases related to it. This should include how the evidence base has been graphically summarized for presentation, and what characteristics were compiled and used to describe the evidence base to readers. |  |
| Risk of bias within individual studies | 12 | Describe methods used for assessing risk of bias of individual studies (including specification of whether this was done at the study or outcome level), and how this information is to be used in any data synthesis. |  |
| Summary measures | 13 | State the principal summary measures (e.g., risk ratio, difference in means). Also describe the use of additional summary measures assessed, such as treatment rankings and surface under the cumulative ranking curve (SUCRA) values, as well as modified approaches used to present summary findings from meta-analyses. |  |
| Planned methods of analysis | 14 | Describe the methods of handling data and combining results of studies for each network meta-analysis. This should include, but not be limited to:   - Handling of multi-arm trials; - Selection of variance structure; - Selection of prior distributions in Bayesian analyses; and - Assessment of model fit. |  |
| **Assessment of Inconsistency** | **S2** | Describe the statistical methods used to evaluate the agreement of direct and indirect evidence in the treatment network(s) studied. Describe efforts taken to address its presence when found. |  |
| Risk of bias across studies | 15 | Specify any assessment of risk of bias that may affect the cumulative evidence (e.g., publication bias, selective reporting within studies). |  |
| Additional analyses | 16 | Describe methods of additional analyses if done, indicating which were pre-specified. This may include, but not be limited to, the following:   - Sensitivity or subgroup analyses; - Meta-regression analyses; - Alternative formulations of the treatment network; and - Use of alternative prior distributions for Bayesian analyses (if applicable). |  |
| **RESULTS** |  |  |  |
| Study selection | 17 | Give numbers of studies screened, assessed for eligibility, and included in the review, with reasons for exclusions at each stage, ideally with a flow diagram. |  |
| **Presentation of network structure** | **S3** | Provide a network graph of the included studies to enable visualization of the geometry of the treatment network. |  |
| **Summary of network geometry** | **S4** | Provide a brief overview of characteristics of the treatment network. This may include commentary on the abundance of trials and randomized patients for the different interventions and pairwise comparisons in the network, gaps of evidence in the treatment network, and potential biases reflected by the network structure. |  |
| Study characteristics | 18 | For each study, present characteristics for which data were extracted (e.g., study size, PICOS, follow-up period) and provide the citations. |  |
| Risk of bias within studies | 19 | Present data on risk of bias of each study and, if available, any outcome level assessment. |  |
| Results of individual studies | 20 | For all outcomes considered (benefits or harms), present, for each study: 1) simple summary data for each intervention group, and 2) effect estimates and confidence intervals. Modified approaches may be needed to deal with information from larger networks. |  |
| Synthesis of results | 21 | Present results of each meta-analysis done, including confidence/credible intervals. In larger networks, authors may focus on comparisons versus a particular comparator (e.g. placebo or standard care), with full findings presented in an appendix. League tables and forest plots may be considered to summarize pairwise comparisons. If additional summary measures were explored (such as treatment rankings), these should also be presented. |  |
| **Exploration for inconsistency** | **S5** | Describe results from investigations of inconsistency. This may include such information as measures of model fit to compare consistency and inconsistency models, P values from statistical tests, or summary of inconsistency estimates from different parts of the treatment network. |  |
| Risk of bias across studies | 22 | Present results of any assessment of risk of bias across studies for the evidence base being studied. |  |
| Results of additional analyses | 23 | Give results of additional analyses, if done (e.g., sensitivity or subgroup analyses, meta-regression analyses, alternative network geometries studied, alternative choice of prior distributions for Bayesian analyses, and so forth). |  |
| **DISCUSSION** |  |  |  |
| Summary of evidence | 24 | Summarize the main findings, including the strength of evidence for each main outcome; consider their relevance to key groups (e.g., healthcare providers, users, and policy-makers). |  |
| Limitations | 25 | Discuss limitations at study and outcome level (e.g., risk of bias), and at review level (e.g., incomplete retrieval of identified research, reporting bias). Comment on the validity of the assumptions, such as transitivity and consistency. Comment on any concerns regarding network geometry (e.g., avoidance of certain comparisons). |  |
| Conclusions | 26 | Provide a general interpretation of the results in the context of other evidence, and implications for future research. |  |
| **FUNDING** |  |  |  |
| Funding | 27 | Describe sources of funding for the systematic review and other support (e.g., supply of data); role of funders for the systematic review. This should also include information regarding whether funding has been received from manufacturers of treatments in the network and/or whether some of the authors are content experts with professional conflicts of interest that could affect use of treatments in the network. |  |

PICOS, population, intervention, comparators, outcomes, study design.

**Supplementary Table 2.** Search strategy

**a:** Search strategy in PubMed

| # | Query |
| --- | --- |
| #1 | " esophageal squamous cell carcinoma”[mh] |
| #2 | esophageal cancer[tiab] OR carcinoma of esophagus[tiab] OR esophageal carcinoma[tiab] OR esophagus cancer[tiab] OR oesophageal cancer[tiab] OR carcinoma of oesophageal[tiab] OR oesophageal carcinoma[tiab] OR oesophageal cancer[tiab] OR EC[tiab] |
| #3 | #1 OR #2 OR |
| #4 | "Neoadjuvant Chemotherapy"[mh] |
| #5 | Neoadjuvant Chemotherapy[tiab] OR Chemotherapy, Neoadjuvant[tiab] OR Neoadjuvant Chemotherapies[tiab] OR Chemotherapy Treatment, Neoadjuvant[tiab] OR Preoperative Chemotherapy[tiab] OR Pre-operative Chemotherapy[tiab] OR Induction Chemotherapy[tiab] |
| #6 | “Immunotherapy”[mh] |
| #7 | Immunotherapies[tiab] OR Programmed death ligand 1[tiab] OR PD-L1[tiab] OR Programmed death 1[tiab] OR PD-1[tiab] OR Anti-Programmed death ligand 1[tiab] OR Anti–PD-L1[tiab] OR Anti-Programmed death 1[tiab] OR Anti–PD-1[tiab] OR Atezolizumab[tiab] OR Durvalumab[tiab] OR Nivolumab[tiab] OR Pembrolizumab[tiab] OR Camrelizumab[tiab] OR Sintilimab[tiab] OR Cemiplimab[tiab] OR Tislelizumab[tiab] OR Ipilimumab[tiab] OR Avelumab[tiab] OR tremelimumab[tiab] |
| #8 | #4 OR #5 OR #6 OR #7 |
| #9 | Randomized Controlled Trial[pt] |
| #10 | Controlled Clinical Trial[pt] |
| #11 | Randomized[tiab] |
| #12 | Placebo[tiab] |
| #13 | Randomly[tiab] |
| #14  #15 | Trial[tiab]  Drug Therapy[sh] |
| #16 | Groups[tiab] |
| #17 | #9 OR #10 OR #11 OR #12 OR #13 OR #14 OR #15 OR #16 |
| #18 | Animals[mh] |
| #19 | Humans[mh] |
| #20 | #20 NOT #21 |
| #21 | #19 NOT #22 |
| #22 | #3 AND #8 AND #21 |

**b:** Search strategy in Embase

| # | Query |
| --- | --- |
| #1 | ‘esophageal squamous cell carcinoma’/exp |
| #2 | ‘esophageal cancer’/exp |
| #3 | 'esophageal squamous cell carcinom':ab,ti OR ‘EC:ab,ti |
| #4 | #1 OR #2 OR #3 |
| #5 | ‘immunotherapy'/exp |
| #6 | 'neoadjuvant chemotherapy'/exp |
| #7 | ‘programmed death ligand 1’:ab,ti OR ‘PD-L1’:ab,ti OR ‘programmed death 1’:ab,ti OR ‘PD-1’:ab,ti OR ‘anti-programmed death ligand 1’:ab,ti OR ‘anti–PD-L1’:ab,ti OR ‘anti-programmed death 1’:ab,ti OR ‘anti–PD-1’:ab,ti OR ‘atezolizumab’:ab,ti OR ‘durvalumab’:ab,ti OR ‘nivolumab’:ab,ti OR ‘pembrolizumab’:ab,ti OR ‘camrelizumab’:ab,ti OR ‘sintilimab’:ab,ti OR ‘cemiplimab’:ab,ti OR ‘avelumab’:ab,ti OR ‘tislelizumab’:ab,ti OR ‘anti–cytotoxic T-lymphocyte antigen 4’:ab,ti OR ‘anti–CTLA-4’:ab,ti OR ‘ipilimumab’:ab,ti OR ‘tremelimumab’:ab,ti OR ‘immunotherapy’:ab,ti OR ‘immune checkpoint inhibitors’:ab,ti OR ‘ICI’:ab,ti |
| #8 | 'preoperative chemotherapy':ab,ti OR ‘neoadjuvant chemotherapy’:ab,ti OR ‘chemotherapy, neoadjuvant’:ab,ti OR ‘neoadjuvant chemotherapies’:ab,ti OR ‘pre-operative chemotherapy’:ab,ti OR ‘induction chemotherapy’:ab,ti |
| #9 | #5 OR #6 OR #7 OR #8 |
| #10 | 'trial':ab,ti |
| #11 | 'random*':ab,ti |
| #12 | 'clinical trial'/de OR 'controlled clinical trial'/de OR 'randomized controlled trial'/de |
| #13 | #10 OR #11 OR #12 |
| #14 | #4 AND #9 AND #13 |

**c:** Search strategy in Cochrane Library

| # | Query |
| --- | --- |
| #1 | MeSH descriptor: [Carcinoma, esophageal] explode all trees |
| #2 | MeSH descriptor: ['esophageal squamous cell carcinoma] explode all trees |
| #3 | ((esophageal OR oesophageal) AND (neoplas* OR cancer OR carcinoma* OR tumour* or tumor*)) |
| #4 | #1 OR #2 OR #3 OR |
| #5 | MeSH descriptor: [Immunotherapy] explode all trees |
| #6 | (programmed death ligand 1 OR PD-L1 OR programmed death 1 OR PD-1 OR anti-programmed death ligand 1 OR anti–PD-L1 OR anti-programmed death 1 OR anti–PD-1 OR atezolizumab OR durvalumab OR nivolumab OR pembrolizumab OR camrelizumab OR sintilimab OR cemiplimab OR avelumab OR tislelizumab OR anti–cytotoxic T-lymphocyte antigen 4 OR anti–CTLA-4 OR ipilimumab OR tremelimumab OR immunotherapy OR immune checkpoint inhibitors OR ICI):ti,ab |
| #7 | (preoperative chemotherapy OR neoadjuvant chemotherapy OR chemotherapy, neoadjuvant OR neoadjuvant chemotherapies OR pre-operative chemotherapy OR induction chemotherapy):ti,ab |
| #8 | #5 OR #6 OR #7 |
| #9 | #4 AND #8 |

**d:** Search strategy in Web of Science

| # | Query |
| --- | --- |
| #1 | TS=(("esophageal" OR "oesophageal") AND (neoplas* OR cancer OR carcinoma* OR tumour* or tumor*)) |
| #2 | TS=("neoadjuvant Chemo*” OR “preoperative chemotherapy” OR “neoadjuvant chemotherapy” OR “chemotherapy, neoadjuvant” OR “neoadjuvant chemotherapies” OR “pre-operative chemotherapy” OR “induction chemotherapy”) |
| #3 | TS=(“programmed death ligand 1” OR “PD-L1” OR “programmed death 1” OR “PD-1” OR “anti-programmed death ligand 1” OR “anti–PD-L1” OR “anti-programmed death 1” OR “anti–PD-1” OR “atezolizumab” OR “durvalumab” OR “nivolumab” OR “pembrolizumab” OR “camrelizumab” OR “sintilimab” OR “tislelizumab” OR “cemiplimab” OR “avelumab”) |
| #4 | TS=(“anti–cytotoxic T-lymphocyte antigen 4” OR “anti–CTLA-4” OR “ipilimumab” OR “tremelimumab”) |
| #5 | TS=(“immunotherapy” OR “immune checkpoint inhibitors” OR “ICI”) |
| #6 | #2 OR #3 OR #4 OR #5 |
| #7 | TS=("randomized controlled trial" OR "controlled clinical trial" OR "clinical trial" OR "random*" OR "rct*" OR "crossover" OR "masked” OR “blind*" OR "placebo*") |
| #8 | #1 AND #6 AND #7 |

**Supplementary Table 3.** Characteristics of studies examining neoadjuvant immune checkpoint inhibitor (ICI).

| First author/Year | Phase (design) | Sample | Male (%) | Median age | Stage 1/2/3/4(%) | ICI intervention*  No.of cycles | Chemotherapy regimen | Surgery  time |
| --- | --- | --- | --- | --- | --- | --- | --- | --- |
| Peng Yang/2021 | prospective study | 16 | 87.5 | 60.5 | 0/25.0/62.5/12.5 | Camrelizumab*2cycles | Paclitaxel plus carboplatin | 4 weeks |
| Xiaolong Yan/2022 | phase II study (TD-NICE) | 45 | 60.0 | 63.8 | 0/40.0/53.3/6.7 | Tislelizumab*3cycles | Carboplatin and albumin-bound paclitaxel | 21-44 days |
| Dijian Shen/2021 | prospective study | 28 | 96.4 | 62.2 | 0/14.3/75.0/10.7 | Nivolumab or pembrolizumab or camrelizumab*2cycles | Paclitaxel and carboplatin | 3–5 weeks |
| Zhigang Wu/2021 | prospective study | 38 | 94.7 | 61 | 0/0/76.3/23.7 | Camrelizumab,  pembrolizumab, or sintilimab*at least 1cycle | Taxel and platinum doublet | 2–13 weeks |
| Xiao Ma/2022 | retrospective study | 34 | 91.2 | 61 | 0/0/41.2/58.8 | Pembrolizumab  or camrelizumab *2cycles | Paclitaxel and cisplatin | 14 weeks |
| Chengqiang Li/2021 | prospective study | 20 | 95 | 62 | 0/10/65/25 | Pembrolizumab | Carboplatin and paclitaxel | 4–6 weeks |
| Jun Liu/2022 | phase II study | 60 | 83.3 | 65 | 0/0/85.0/15.0 | Camrelizumab | Nab-paclitaxel and carboplatin | 4–6 weeks |
| Weixiong Yang/2021 | prospective study | 23 | 95.7 | 58.6 | 0/34.8/65.2/0 | Camrelizumab | Nab-paclitaxel and carboplatin | 3–6 weeks |
| Bingjiang Huang/2021 | prospective study | 23 | 91.3 | 59.2 | 0/13.0/60.9/26.1 | Pembrolizumab*2cycles | Docetaxel and nidaplatin | 4–6 weeks |
| Zhinuan Hong/2021 | retrospective study | 38 | 57.9 | 58.8 | 50.0/10.5/39.5/0 | Camrelizumab*(2~4) cycles | Paclitaxel | 4–8 weeks |
| Guozheng Yang/2021 | retrospective study | 12 | 58.3 | 56 | 0/16.7/66.6/16.7 | Camrelizumab | Nab-paclitaxel/S1 | 0.4-2.9 weeks |
| Wenqun Xing/2021 | phase II study | 24 | / | / | / | Toripalimab | Paclitaxel and cisplatin | 4–6 weeks |
| Yimin Gu/2022 | retrospective study | 38 | 71.1 | 66 | 0/23.7/76.3/0 | Pembrolizumab, tislelizumab, camrelizumab, sintilimab and toripalimab*(2~4) cycles | Paclitaxel plus cisplatin | 40-147 days |
| Yehan Zhou/2022 | retrospective study | 14 | 64.3 | / | / | Toripalimab*2 cycles | Paclitaxel plus carboplatin | 22-36 days |
| Jianhua Liu/2022 | prospective study | 37 | / | / | / | Tirelizumab, pembrolizumab | Nab-paclitaxel and cisplatin | / |
| Jiahan Cheng/2022 | retrospective study | 40 | 75.0 | 64.3 | 0/5.0/92.5/2.5 | Pembrolizumab, tislelizumab, camrelizumab, sintilimab, toripalimab *(2~4) cycles | Paclitaxel plus cisplatin, fluorouracil plus cisplatin | / |
| Zhinuan Hong/2022 | retrospective study | 32 | 65.6 | 62 | / | Sintilimab, pembrolizumab, toripalimab, camrelizumab *(2~4) cycles | Platinum-based drugs  and paclitaxe | / |
| Lei Gao/2022 | phase II study | 20 | 85.0 | 58.3 | / | Toripalimab | Docetaxel and cisplati | 4–6 weeks |
| Shujie Huang/2022 | real-word multicenter study | 155 | 78.1 | 61 | 0/0/13.5/88.5 | Camrelizumab, pembrolizumab, sintilimab, tislelizumab,  toripalimab, and nivolumab | Platinum-based plus docetaxelor taxane-based agents | / |
| Hongtao Duan/2022 | prospective study | 18 | 77.8 | 64 | 0/38.9/55.6/5.6 | Pembrolizumab | Docetaxe plus nedaplatin, nab-paclitaxel plus nedaplatin | 4–6 weeks |
| Yujin Qiao/2022 | retrospective study | 48 | 79.2 | 64.15 | / | Camrelizumab | Paclitaxel, albumin-bound  paclitaxel or docetaxel | / |
| Xiao Xin/2022 | retrospective study | 55 | 83.6 | 66 | 0/14.5/65.5/20.0 | Pembrolizumab, sintilimab, tislelizumab, camrelizumab*2 cycles | Paclitaxel, cisplatin | 6-8 weeks |
| Guoqiang Yin/2022 | retrospective study | 34 | 88.2 | 59 | 0/14.7/76.5/8.8 | Camrelizumab | Carboplatin | 6 weeks |
| Zhenyang Zhang/2021 | phase II study | 30 | 87 | 58.3 | 0/0/90/10 | Sintilimab*2 cycles | Cisplatin | 4–6 weeks |
| Huilai Lv/2022 | prospective study | 96 | 69.8 | 65 | 0/41.7/54.2/4.2 | Sintilimab*(2~4) cycles | Platinum and taxanes | / |
| Wenwu He/2022 | phase II study | 20 | 75.0 | 61.4 | 0/0/80.0/20.0 | Toripalimab*2cycles | Paclitaxel and carboplatin | 6 weeks |
| Jun Liu/2021 | phase II study | 51 | 80.4 | 60 | 0/25.5/64.7/9.8 | Camrelizumab | Nab-paclitaxel and cisplatin | 6 weeks |
